# Unveiling the sp^2^─sp^3^ C─C Polar Bond Induced Electromagnetic Responding Behaviors by a 2D N‐doped Carbon Nanosheet Absorber

**DOI:** 10.1002/advs.202306159

**Published:** 2023-12-03

**Authors:** Can Zhang, Jian‐Tang Jiang, Zhenjie Guan, Yuanyuan Zhang, Yining Li, Bo Song, Wenzhu Shao, Liang Zhen

**Affiliations:** ^1^ School of Materials Science and Engineering Harbin Institute of Technology Harbin 150001 P. R. China; ^2^ National Key Laboratory of Precision Hot Processing of Metals Harbin Institute of Technology Harbin 150001 P. R. China; ^3^ National Key Laboratory of Science and Technology on Advanced Composites in Special Environments Harbin Institute of Technology Harbin 150080 P. R. China; ^4^ Sauvage Laboratory for Smart Materials School of Materials Science and Engineering Harbin Institute of Technology (Shenzhen) Shenzhen 518055 P. R. China

**Keywords:** carbon nanosheets, dielectric behavior, electric dipole, electromagnetic absorption, sp^2^─sp^3^ C─C bond

## Abstract

The infertile electromagnetic (EM) attenuating behavior of carbon material makes the improvement of its performance remain a significant challenge. Herein, a facile and low‐cost strategy radically distinct from the prevalent approaches by constructing polar covalent bonds between sp^2^‐hybridized and sp^3^‐hybridized carbon atoms to introduce strong dipolar polarization is proposed. Through customizing and selectively engineering the N moieties conjugated with carbon rings, the microstructure of the as‐synthesized 2D nanosheet is gradually converted with the partial transition from sp^3^ carbons to sp^2^ carbons, where the electric dipoles between them are also tuned. Supported by the DFT calculations, a progressively enhanced sp^2^─sp^3^ C─C dipolar polarization is caused by this controllable structure evolution, which is demonstrated to contribute dominantly to the total dielectric loss. By virtue of this unduplicated loss behavior, a remarkable effective absorption bandwidth (EAB) beyond ‐10 dB of 8.28 GHz (2.33 mm) and an ultrawide EAB beyond ‐5 dB of 13.72 GHz (4.93 mm) are delivered, which upgrade the EM performance of carbon material to a higher level. This study not only demonstrates the huge perspective of sp^2^─sp^3^‐hybridized carbon in EM elimination but also gives pioneering insights into the carbon–carbon polarization mechanism for guiding the development of advanced EM absorption materials.

## Graphical abstract

The sp^2^─sp^3^‐hybridized 2D carbon nanosheets are synthesized via a facile and low‐cost salt‐assisted thermal‐polymerization and pyrolysis strategy. The materials composed of only C and N atoms present ultrawide electromagnetic absorption bandwidth. This study excitingly reveals that sp^2^─sp^3^ C─C polar covalent bonds enable strong dipolar polarization loss.

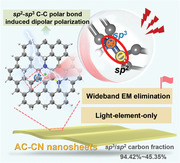

## 1 Introduction

The advent of fifth‐generation (5G) wireless communication represents a significant leap forward in human technology, unfortunately, undesirable electromagnetic (EM) interference and pollution are there of generated triggering negative effects on the environment and people's health.^[^ ^1^, ^2^, ^3^, ^4^ ^]^ In response to these issues, a multitude of EM absorption materials have been developed over the years, among which carbon stands out as a particularly promising candidate for its practical merits, such as lightweight, physicochemical stability, and high tunability.^[^ ^5^ ^]^ However, challenges emerge due to the limited attenuation capacity of bare carbons, which causes their narrow EM responding frequency range. To this end, a wide range of strategies–including morphology design,^[^ ^6^ ^]^ component modulation,^[^ ^7^ ^]^ and interface/defect/doping engineering^[^ ^8^, ^9^, ^10^, ^11^ ^]^ –have been exploited with the aim of introducing the synergistic lossy effects and thus achieving stronger dielectric loss.

As is well‐known, doping engineering, represented by N doping, is capable of adjusting the electronic properties of carbon atoms to modulate the EM behaviors. Both experiments and theoretical calculations show that permanent electric dipoles are developed when N atoms are doped into the carbon lattice framework.^[^ ^12^ ^]^ Excessive attention was paid to the heteroatom edge sites (e.g., pyrrolic‐N and pyridinic‐N) which are believed to play a key role in generating dipolar polarization due to their ability to afford in‐plane defects throughout the sp^2^ carbon rings. Boosted EM performance has so far been realized via N doping in various carbon models such as graphene, nanotubes, and foams.^[^ ^13^, ^14^, ^15^ ^]^ Nevertheless, despite these impressive advances, a bridge linking the microstructure with the dielectric loss for the N doping in carbon at the atomic scale has yet to be established. Particularly, little in‐depth attention has been given to the impact of the N species’ regulation accompanied by the carbon structure evolution on its dielectric loss behaviors. Actually, the dipole moment effect induced by N doping is not only dependent on the intrinsic properties of bond dipole moments but also sensitive to the surrounding chemical environment. Considering these problems, customizing and selectively engineering N moieties within the carbon matrix is thus of paramount significance in revealing the dielectric loss mechanism in N‐doped carbon absorbers.

More importantly, previous studies on carbon‐based EM absorbers excessively focused on the sp^2^‐hybridized carbons since the formation of sp^2^ carbon is much more thermodynamically and technically accessible compared to that of sp^3^‐hybridized carbon. The nature of high electric conductivity for sp^2^ carbons is leveraged to induce resistance loss by constructing localized conductive networks.^[^ ^16^ ^]^ In traditional nanocarbon models, resistance loss overwhelmingly dominates the dielectric loss, by which a strong reflection loss (RL) over a narrow bandwidth can be aroused. For example, RLs of −45.5 and −51.1 dB were delivered by the sp^2^‐hybridized graphene/carbon nanotubes hybrid and the graphene nanocages systems, respectively, while the effective absorption bandwidths (EAB) were limited to only 5.6 and 4.40 GHz in these researches.^[^ ^17^, ^18^ ^]^ By contrast, the EM response of sp^3^ carbon has barely been studied yet. The differences in intrinsic electronic properties between sp^2^ carbon and sp^3^ carbon, especially in electronegativity, are able to introduce interesting EM responding effects to a sp^2^─sp^3^‐hybridized C─C system. Based on fundamental polarization theory, a bond between two carbon atoms of different hybridization can be polarized,^[^ ^19^ ^]^ indicating that the C─C electric dipole moments spontaneously form between sp^2^ and sp^3^ carbons. Meanwhile, as a defective role in the sp^2^ framework, sp^3^ carbon with tetrahedral orbitals will automatically introduce severe lattice distortion of the carbon skeleton to strengthen the electric dipoles. In this direction, a different model compared to the state‐of‐the‐art EM absorbers can be conceptually proposed here, in which, instead of sp^2^ carbon atoms as the electrical conductor, sp^2^─sp^3^ bonded carbon structures are designed as dipolar polarization centers. This means a distinctive strategy of constructing dipoles between atoms of the same element as a promising solution to the extension of the EM responding bandwidth of carbon materials. Meanwhile, difficulties still lie in the implantation of sp^3^ carbons into the sp^2^ carbon matrix since the formation of sp^3^ carbon generally involves harsh conditions such as high temperature and high pressure.^[^ ^20^, ^21^, ^22^ ^]^ Therefore, it is extremely significant and intriguing to explore the sp^2^‐sp^3^ C─C dipole‐induced polarization behaviors and to excavate their potential in attenuating EM energy as long as a facile approach is developed.

In this study, we present a type of sp^2^─sp^3^‐hybridized 2D carbon nanosheet (AC‐CN) synthesized via a facile and low‐cost salt‐assisted thermal‐polymerization and pyrolysis strategy. The presence of high‐content sp^3^ carbons exerts a momentous impact on the electronic property and the dielectric behaviors of the sp^2^ carbon matrix. By adjusting the pyrolysis temperature, the AC‐CN nanosheets transition from a carbon rings‐triazine units conjugated heterostructure to a selectively engineered N‐doped carbon structure, which simultaneously tunes sp^2^ and sp^3^ carbons and the polar bond between them. Supported by DFT theoretical calculations, the mechanistic insights into the electronic interactions and polarization behaviors of sp^2^─sp^3^ hybridization evolution were deeply uncovered at the atomic level. For the first time, our findings excitingly demonstrate that the electric dipoles induced by the polar covalent bond between sp^2^─sp^3^ C─C are able to engender strong polarization loss, by virtue of which AC‐CN nanosheet with an optimized sp^3^/sp^2^ carbon ratio delivers remarkable EM performance. Specifically, the EAB_10_ (beyond ‐10 dB) of 8.28 GHz at 2.33 mm and the astonishing EAB_5_ (beyond −22125 dB) of 13.72 GHz at 4.93 mm have upgraded the EM absorption performance of carbon‐based absorbers. This research not only takes the first step toward the exploration of sp^2^─sp^3^‐hybridized carbon for EM absorption but also offers in‐depth insights into the polarization mechanism regarding carbon‐carbon bonds to accelerate the development of novel absorbers.

## 2 Results and Discussion

### 2.1 Design and Synthesis of 2D sp^2^─sp^3^‐Hybridized AC‐CN Nanosheets

**Figure 1:**
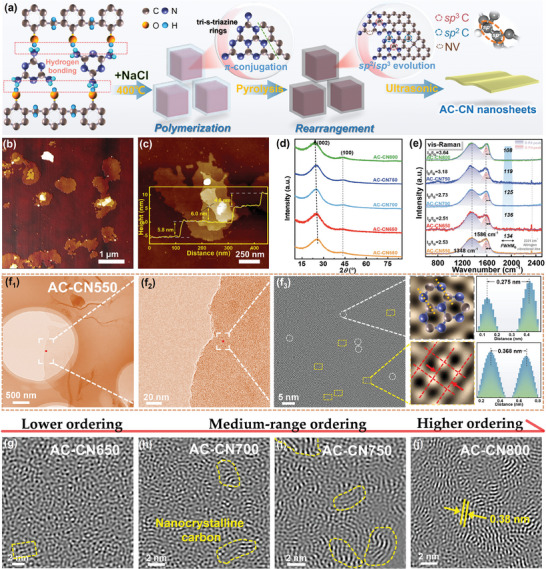
a) Schematic illustration of the synthetic route of AC‐CN nanosheets. b,c) AFM images of AC‐CN750 (the inset reflects the thickness information). d) XRD patterns and e) vis‐Raman spectra of AC‐CN nanosheets. f1,f2) TEM, and f3) inverse Fourier transform of the HRTEM images of AC‐CN550 (The elliptic area is the tri‐s‐triazine domain, and the rectangular area is the randomly oriented SRO cluster). g–j) Inverse Fourier transform of the HRTEM images of AC‐CN650, AC‐CN700, AC‐CN750, and AC‐CN800, respectively.

A salt‐assisted thermal‐polymerization and pyrolysis route was developed to synthesize 2D sp^2^─sp^3^‐hybridized AC‐CN nanosheets as depicted schematically in **Figure** **1**a. Due to the similar aromatic tecton and the lone‐pair‐electron‐rich N sites at the edge of triazine rings, resol molecules, and triazine units are prone to undergo a dehydration reaction under the moderate thermal condition of 400 °C and form a planar conjugated structure (carbon rings‐triazine units) via C─N bonds.^[^ ^23^ ^]^ The electronic configuration of the carbon atoms at the coalescence sites will convert from sp^2^ hybridization to sp^3^ hybridization because of the cleavage of π bond, which directly leads to the formation of sp^2^─sp^3^ C─C polar covalent bonds. When calcined at high temperatures, the carbon domains were further carbonized whilst the triazine domains gradually decomposed with the sp^2^─sp^3^ C─C partially preserved, allowing for selective modulation of the sp^2^─sp^3^ carbon ratio.

In these processes, NaCl crystals provide platforms for their polymerization along the in‐plane direction and thereby ensure the 2D structure of the product, as demonstrated by the atomic force microscopy (AFM) images (Figure 1b). The as‐prepared AC‐CN nanosheets show a size of micrometer scale and a small thickness of ≈5‐6 nm (Figure 1c). Both the XRD patterns and visible Raman (vis‐Raman) spectra reveal their structural nature of low graphitization degree (Figure 1d,e). Of note in vis‐Raman spectra, G bands that arise from the in‐plane stretching motion of *E*_2g_ symmetry of sp^2^ pairs of carbon show an evidently strengthened dispersion compared to that of the control sample AC750 (Figure S1, Supporting Information), which is indicative of the defective structure induced by the incorporation of sp^3^ carbons.^[^ ^24^ ^]^ A continuously enhanced ordering degree of carbon framework is also disclosed by the decreasing dispersion of G band upon the elevated pyrolysis temperature. Furthermore, the morphology and the structural evolution were observed via transmission electron microscopy (TEM) and high‐resolution TEM (HRTEM), as presented in Figure 1f. In conjunction with the high‐angle annular dark field (HADDF) images (Figure S2, Supporting Information), the bright‐field TEM images reveal that the as‐synthesized AC‐CN nanosheets comprise several layers of delicate and thinner nanosheets stacked together, exhibiting a smooth and seamless surface without presence of any discernible nanoholes or nanoparticles but with certain curvature. An inverse Fourier transform of the HRTEM image demonstrates the highly disordered microstructure of AC‐CN550, as previously unraveled by XRD and vis‐Raman results, in which several different lattice fringe regions are found (Figure 1f_3_). Thereinto, the lattice fringe with an in‐plane spacing of ≈0.275 nm could be assigned to the triazine domain, the presence of which has been well demonstrated by FTIR spectra (Figure S3, Supporting Information). In the meantime, it is noted that loads of short‐range ordered (SRO) clusters with the “crystal‐plane‐like” stripes of ≈90° intersecting angles are randomly incorporated in the disordered plane. The region shows a local structural characteristic quite similar to that of the diamond but with a larger lattice distance of ≈0.368 nm, which intuitively indicates that the sp^3^ carbon cluster is presumably formed within the carbon backbone. Also, energy dispersive X‐ray spectroscopy (EDS) mapping indicates the homogeneous distribution of C and N atoms throughout the nanosheet (Figure S4, Supporting Information). With the increasing pyrolysis temperature, the 2D sheet‐like morphology of the samples remains consistent throughout; nevertheless, a gradual transformation toward higher ordering in microstructure can be observed. For AC‐CN650, a similar disordered structure to that of AC‐CN550 implies that essential structural alterations cannot be aroused under the fairly mild temperature (Figure 1g), inconsistent with the results of vis‐Raman spectra. Also of note is that the cluster of sp^3^ carbon is sharply decreased in AC‐CN650 due to the degradation of N moieties. As the pyrolysis temperature increases from 700 to 750 °C, some nanocrystalline carbon structures with medium‐range ordering emerge increasingly, suggesting enhanced stability of the carbon skeleton (Figure 1h,i). Moreover, raising the pyrolysis temperature to 800 °C further gives rise to a higher ordering of sp^2^ carbon domains, in which the much clearer lattice fringe with a spacing of 0.38 nm, corresponding to the (100) plane of graphitic carbon, can be observed (Figure 1j). Meanwhile, the gradual growth of the sp^2^ nanocrystalline also well explains the shift of (002) diffraction peaks toward smaller degrees as detected in XRD patterns.

### 2.2 Structural Evolution of AC‐CN Nanosheets via Selectively Engineering N Moieties

**Figure 2:**
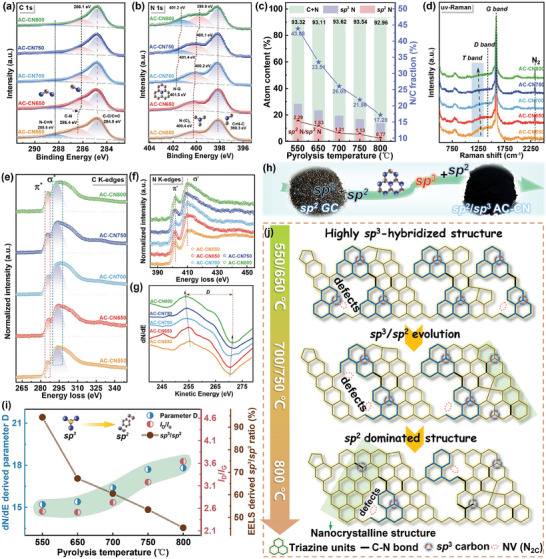
High‐resolution XPS spectra of a) C 1s and b) N 1s. c) Atomic content, N/C fraction and sp^2^ N/sp^3^ N ratio derived from XPS. d) uv‐Raman spectra. STEM‐EELS spectra of e) C K‐edges and f) N K‐edges fine structures, respectively. g) XAES spectra of C KLL. h) Digital images showing the appearance change from bare AC750 (left) to AC‐CN750 (right). i) The variation of parameter D, *I*_D_/*I*_G_, and sp^3^/sp^2^ carbon fraction of AC‐CN nanosheets with the pyrolysis temperature. j) Schematic illustration of the microstructure evolution of AC‐CN nanosheets.

X‐ray photoelectron spectrum (XPS) was exploited to further elucidate the structural evolution. Considering the small thickness (≈5‐6 nm) of AC‐CN nanosheets, the XPS technique is able to reflect the in‐building chemical states in addition to those of the surface. The results show that C and N atoms dominate the composition with the presence of a small content of O atoms (Figure S5, Supporting Information). In specific, the high‐resolution spectra of C 1s are deconvoluted into three peaks assigned to C─C/C═C at 284.8 eV, C─N at 286.4 eV, and N─C═N at 288.6 eV, respectively (**Figure** **2**a).^[^ ^25^, ^26^ ^]^ Besides, the N 1s spectra present two respective peaks at 398.3 and ∼400 eV corresponding to sp^2^‐hybridized bi‐coordinated C═N─C (N_2C_) and sp^3^‐hybridized tertiary N─(C)_3_, which are in good consistent with the N configurations from tri‐s‐triazine (Figure 2b).^[^ ^27^, ^28^, ^29^, ^30^ ^]^ However, as the pyrolysis temperature increases up to 700 °C, a new peak indexed to graphitic‐N (N‐Q) appears at 401.5 eV,^[^ ^31^ ^]^ indicating a localized microstructural transformation from carbon rings‐triazine units conjugated plane toward N doped carbon skeleton with a higher structural stabilization. Actually, the slight shifts to lower binding energies (0.3 eV) of the C─N bond in C 1s spectra for the AC‐CN nanosheets prepared above 650 °C has witnessed the decomposition of triazine units as the result of its thermal instability beyond 600 °C. In addition, the gradient shift of N─(C)_3_ toward lower binding energy in XPS spectra of N 1s is ascribed to the formation of adjacent N vacancy (NV), i.e., N_2C_ vacancies, during the decomposition of triazine when the pyrolysis temperature is elevated to 700 °C.^[^ ^32^, ^33^ ^]^ Based on the XPS data, the N/C fraction in AC‐CN nanosheets is found to decrease from 43.88% to 17.20% accompanying with a reduction of sp^2^ N/sp^3^ N ratio from 2.29 to 0.77 (Figure 2c). This is not only an indicative sign of the N species’ modulation accompanied by the structure evolution of the carbon skeleton, but also a valid demonstration showing that N_2C_ sites are preferentially removed in triazine rings under pyrolysis. By this means selectively engineering the N‐containing group is successfully realized. Meanwhile, as shown in Figure 2h, one interesting phenomenon of note is that the as‐obtained AC‐CN nanosheet powder shows a distinct appearance with the absence of glassy luster and a visibly finer carbon particle in contrast to AC750, indicating the magnificent differences in their microstructure aroused by the introduction of N species and sp^3^ carbons (Figure S6, Supporting Information). The presence of sp^3^ carbon can be directly detected by the ultraviolet resonant Raman (uv‐Raman) spectrum, as there exists a positive correlation between f the Raman signal intensity and the fourth power of the excitation frequency. Distinct from vis‐Raman spectra, the uv‐Raman spectra in Figure 2d present a sharp G band at ≈1592 cm^−1^ with high intensity for all samples, while the shoulder‐like D band becomes nearly invisible in AC‐CN700/750/800 nanosheets, which agrees with their structural evolution with the formation of nanocrystalline and reveals the stronger Raman signals from sp^2^ carbon vibrations. More importantly, a broad hill‐shaped peak at ≈1250 cm^−1^, namely the T band, is also exhibited by AC‐CN nanosheets. The T band originates from the vibrations of *σ* states, sensitive only to sp^3^ C hybridization.^[^ ^34^, ^35^ ^]^ For this reason, the control sample AC750 shows no T band in its uv‐Raman spectrum but still presents an evident D band together with an ultrahigh G band (Figure S7, Supporting Information). By contrary, the T band exhibited by the as‐synthesized AC‐CN nanosheets preliminarily demonstrates the presence of polar covalent bonds between sp^2^ and sp^3^ carbons in these structures. Additionally, the fact that sp^3^ carbons are partially converted into sp^2^ carbons upon the elevated pyrolysis temperature is unveiled through the gradual decrease in the intensity of T bands.

The sp^2^─sp^3^ hybridized structure was further investigated using scanning transmission electron microscopy and electron energy loss spectroscopy (STEM‐EELS), from which the homogeneous distribution of C and N atoms is well defined (Figure S8, Supporting Information). For the as‐synthesized AC‐CN nanosheets, the electron energy‐loss near‐edge structure (ELNES) acts as the unique fingerprints to track sp^2^ and sp^3^ carbons by revealing the local density of unoccupied states. As seen in Figure 2e, the C‐K edge EELS core‐loss spectra show two characteristic features: the 1s→anti‐bonding *π* ^*^ transition peak at ≈286 eV and the 1s→anti‐bonding *σ* ^*^ transition peak centered at ≈293 eV. Based on the intensity integration of two energy windows (284.0–288.0, 288.5–298.5 eV), the relative content of sp^3^ carbon was evaluated by using the fully sp^2^‐hybridized glass carbon (GC) as the standard reference.^[^ ^36^, ^37^ ^]^ After calculation, it is demonstrated that the sp^3^/sp^2^ fraction of carbon atoms monotonously decreases with the increasing pyrolysis temperature (as listed in Table S1, Supporting Information). These results quantitatively provide definitive evidence for the gradual transition of carbon configuration from sp^3^ to sp^2^. The highest content of sp^3^ carbon (sp^3^/sp^2^ fraction ≈94.42%) for AC‐CN550 explains its strongest T band in the uv‐Raman spectra, and even for the sample AC‐CN800 prepared under the highest temperature, there remains sizeable sp^3^/sp^2^ carbon ratio up to ≈45.35%. Such a high atomic content means that sp^3^ C atoms can be massively preserved under the higher pyrolysis temperature regardless of the loss of N species. Moreover, similar spectra with two peaks located at ≈402 and ≈409 eV are displayed by the N‐K edge fine structure (Figure 2f), further demonstrating the presence of sp^2^ bonded N as well as the carbon rings‐triazine units conjugated structure. The atomic fraction of N/C calculated from EELS is in good line with that from XPS (Table S2, Supporting Information)). In this regard, one can find that the removal of N atoms is much more sensitive to the increasing pyrolysis temperature than the conversion of sp^3^ C. The X‐ray Auger‐electron spectroscopy (XAES) also gives a photoelectronic fingerprint of the chemical state for demonstrating the evolution of sp^2^ bonding toward sp^3^ bonding (Figure S9, Supporting Information).^[^ ^38^, ^39^ ^]^ As seen, the derivative dN/dE spectra derived from C KLL XAES spectra show a changing parameter *D_KEW_* (kinetic energy width) between the maximum of the positive‐going excursions and the minimum of the negative‐going excursions (Figure 2g). Notably, the *D_KEW_* value exhibits a basically synchronous enhancement with *I*_D_/*I*_G_ of Raman spectra upon the elevated temperature (Figure 2i). It is well‐established that the D band arising from the breathing mode of *A*_1g_ symmetry in sp^2^ six‐fold aromatic rings represents the lattice disorder, thus the ratio of *I*_D_/*I*_G_ is usually exploited to reflect the ordering degree based on the well‐known Tuinstra and Koenig (TK) relationship. However, for the as‐obtained AC‐CN nanosheets with both the abundant sp^3^‐hybridized and nanocrystalline structure, the D‐band intensity actually indicates the probability of finding a six‐fold ring in the nanocrystalline cluster, which is proportional to the cluster size. In this situation, the TK relation is no longer applicable. Therefore, the equation proposed by Ferrari and Robertson, $I_{D}/I_{G}=C^{\prime }\;\left(\lambda \right)L_{a}^{2}$, to apply for the disordered structure between nanocrystalline graphite and sp^3^‐containing amorphous carbon can effectively interpret the enhanced *I*_D_/*I*_G_ ratio of AC‐CN nanosheets with rising temperature;^[^ ^34^ ^]^ that is to say, the enlarged nanocrystalline structure, as observed in HRTEM, is responsible for the elevated *I*_D_/*I*_G_ ratio.

Based on the above results, the structural evolution of AC‐CN nanosheets is fully validated as involving the loss or transition of N species and sp^2^─sp^3^ carbons. The evolutionary mechanism behind the selectively engineered sp^2^─sp^3^‐hybridized carbon structure can be summarized in three processes, as illustrated in Figure 2j,i) The initial highly disordered heterostructure consisting of localized sp^3^ carbon clusters and triazine domains becomes unstable under temperatures beyond 600 °C due to the decomposition of triazine; ii) The carbon nanocrystalline is formed accompanying the formation of selectively induced N_2C_ NVs and the decreasing fraction of sp^3^/sp^2^ carbons under 700 °C, corresponding to a gradually stabilized structure; iii) Finally, a sp^2^‐carbon dominated structure of highest ordering degree is obtained with the ongoing detachment of N moieties and the rearrangement of C under the pyrolysis temperature of 800 °C. Therefore, as we expected, the construction of a sp^3^‐ridden carbon hybrid framework is realized by the customization and the in situ conversion of the carbon skeleton during the pyrolysis process. Based on the results above, the related dielectric behaviors will be carefully explored in conjunction with the elaborate microstructure of AC‐CN nanosheets in the following discussion.

### 2.3 sp^2^─sp^3^ C─C Polar Covalent Bonds Induced Dipolar Polarization Behavior

**Figure 3:**
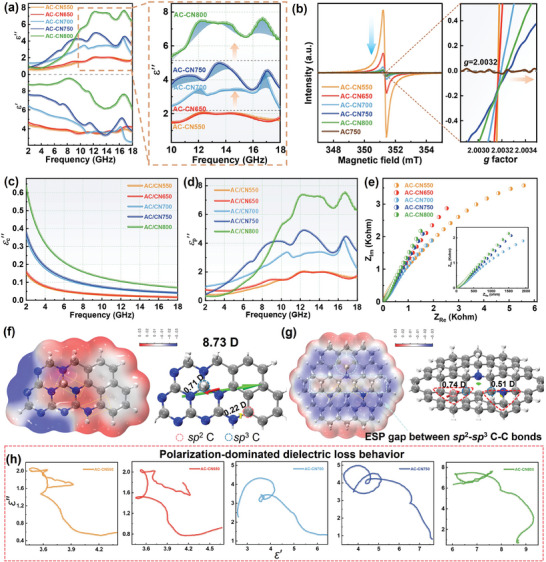
a) The frequency dependence of ε′ and ε′′, b) EPR spectra, c,d) Fitted *εc′*′ and *εp′′* curves, and e) Nyquist plots of AC‐CN nanosheets. ESP map (left) and the electric dipoles (right) obtained by DFT simulation of f) the carbon rings‐triazine units conjugated structure, and g) the more ordered hybrid structure with sp3 carbons incorporated within the sp2 domain (gray ball for C atom, blue ball for N atom). h) Cole–Cole plots of AC‐CN nanosheets.

The EM parameters were investigated as the important linkage between the EM responding modes and the evolution of AC‐CN nanosheets’ microstructure in the frequency range of 2–18 GHz. Considering the negligible magnetic properties of the as‐prepared carbon systems (Figure S10 Supporting Information), the dielectric loss that is reflected by the real part (*ε*′) and the imaginary part (*ε*′′) of complex permittivity thereby decidedly represents their EM responding behaviors. First, we notice that multiple relaxation peaks are exhibited by AC‐CN nanosheets (**Figure** **3**a; Figure S11, Supporting Information). For AC‐CN550 and AC‐CN650, their permittivities present a very analogous dependence on the frequency with fluctuations in a small range due to the indistinguishable differences in their microstructure, where *ε*′ slightly decreases from ≈4.4 to ≈3.7 while *ε*′′ varies progressively from ≈0.6 to ≈1.8. With increasing the pyrolysis temperature to and above 700 °C, the *ε*′ still shows a similar variation trend but with a much more intense frequency dispersion effect over the whole frequency, which implies their strengthened dielectric polarization ability that relates to the upgraded microstructure. It is also noticed that the *ε*′′ curves barely change in the frequency range of ≈2–4 GHz with a low initial value of ≈1.0, followed by a gradual increase and a final fluctuation over the frequency range of ≈10–18 GHz. The low *ε*′′ in the first half frequency (below 10 GHz) range directly indicates the unqualified EM performance in this region. However, the increasingly frequency‐dependent *ε′′* in the second half frequency range (above 10 GHz), which numerically approaches *ε*′, results in the capacity matching between electric‐energy storing and consuming, so as to provide a prerequisite for the strong absorption over X and Ku bands. In particular, this phenomenon becomes most prominent in AC‐CN750, reflected by its dielectric loss factor (tan *δ*_E_ = *ε′′*/*ε*′) of ≈1.0 covering the widest frequency band (Figure S11 Supporting Information), revealing that outstanding EM absorption performance can be expected by the highly close *ε*′ and *ε′′*.

The solution to unraveling the underlying EM response mechanism of AC‐CN nanosheets lies in the changing mode of permittivity. First, one can note that compared to bare carbon AC750, the AC‐CN nanosheets show an obviously smaller *ε*′ and a more frequency‐sensitive *ε*′′ concurrently, which strongly suggests that the incorporation of N species and sp^2^─sp^3^ C─C bonds has significantly modified the dielectric loss behaviors. On one hand, the observed increase in *ε*' with frequency can be attributed to the occurrence of polarization relaxations. This is because polarization behaviors such as defect polarization, dipolar polarization, and interfacial polarization are only induced when the EM energy reaches a certain threshold. On the other hand, the electrically conductive ability that positively contributes to *ε*′′ should be taken into account as well in terms of the Debye theory and the free electron theory. Given these concerns, the related EM responding behaviors involving multiple factors seem complicated to analyze. Therefore, further investigation into the dielectric loss modes of AC‐CN nanosheets was conducted by combining a more in‐depth understanding of the structural evolution based on the dielectric properties involved.

Indeed, it is well‐defined that the as‐prepared AC‐CN nanosheets possess an elaborate and complex microstructure, in which vacancies, N species, and sp^2^─sp^3^ bonds coexist. Despite the interactive contribution of these factors to polarization, some evidence for distinguishing the lossy role of them still can be found. We initiate our investigation from the defect‐induced dipolar polarization since the contribution from it is straightforwardly related to the concentration of vacancy for the as‐prepared AC‐CN nanosheets. The obtained characterization results have validated the enhanced ordering degree of AC‐CN nanosheets with the elevated pyrolysis temperature. On this basis, electron paramagnetic resonance (EPR) spectroscopy and photoluminescence (PL) spectrum were performed to provide more direct and detailed information on the defect structure. Zero EPR signal delivered by the bare carbon AC750 reveals its saturated configuration of C atoms (Figure 3b), in consistent with the nearly fully sp^2^‐hybridized and vacancy‐free nature of GC. In comparison, AC‐CN nanosheets show obvious Lorentzian EPR peaks with a basically decreasing intensity, which further confirms the introduction of vacancies into the carbon framework and the reordering of defect‐ridden structure upon the increasing pyrolysis temperature.^[^ ^40^ ^]^ The same conclusion can be drawn from the PL spectra (Figure S12, Supporting Information). The *g* factor of ≈2.0032 reveals that the EPR signal stems from the unpaired electrons of C atoms within the π‐conjugated aromatic rings.^[^ ^41^, ^42^ ^]^ Combining the XPS results, it was further demonstrated that an abundance of NVs is present.^[^ ^43^ ^]^ The introduction of these NVs enables the reallocation of extra electrons to neighboring C atoms via the delocalized π‐conjugated C─N networks.^[^ ^44^, ^45^ ^]^ Also worth noting is that the microstructural evolution toward high ordering develops accompanying with the simultaneous detachment of N_2C_ atoms to form NVs. The formation of N–Q atoms may indicate that the orderly rearrangement of C atoms leads to the replacement of N_2C_ vacancies by C atoms. The results above provide sufficient precondition for the occurrence of defect‐induced dipolar polarization in AC‐CN nanosheets. Nonetheless, the inverse relationship between the decreasing defect concentration and the enhancing *ε*′′ obviously discloses that these defects contribute slightly to the dielectric loss. Hence, the focus needs to be shifted to the polarization behaviors induced by the N dopant and sp^2^─sp^3^ C─C bond. Before this, it is necessary to figure out the contribution of the electrical conductivity (*σ*) on the variation of *ε*′′ because we know that the decrease of defects favors giving rise to an improvement in *σ*.^[^ ^46^ ^]^

The effect of *σ* on *ε*′′ is interpreted in the following formula (*ε*_0_, the relative complex permittivity of free space), from which the contribution of polarization loss and resistance loss can be separately derived through the nonlinear least squares fitting method:^[^ ^47^ ^]^

(1) $$\varepsilon \prime \prime =\varepsilon_{P}\prime \prime +\varepsilon_{c}\prime \prime =\frac{2\pi f\tau \left(\varepsilon_{s}-\varepsilon_{\infty }\right)}{1+{\left(2\pi f\right)}^{2}\tau^{2}}+\frac{\sigma }{2\pi f\varepsilon_{0}}$$

In Figure 3c, the small *ε*_c_′′ presented by the AC‐CN nanosheets unambiguously indicates their weak resistance loss ability, which can be easily anticipated, in fact, by the presence of high‐content sp^3^ carbons. To intuitively disclose the correlation between the microstructure and conductivity, electrochemical impedance spectroscopy (EIS) was conducted, as shown in Figure 3e. A decreasing tendency of charge transfer resistance with the reduction of sp^3^ carbon content is clearly revealed in the fitted Nyquist curves, especially for AC‐CN700/750/800, providing the explanation for the gradually increasing *ε*_c_′′. In this regard, the phenomenon well corresponds to the strengthened frequency dispersion of the AC‐CN nanosheets prepared above 700 °C, suggesting that the electron migration‐related EM loss behaviors cannot be ruled out in these samples. This can be attributed to the formation of both the localized sp^2^ carbon nanocrystalline that functionally affords a path for electron transmission and N‐Q toms that induce a higher charge delocalization in these samples. Nevertheless, the lossy contribution from resistance loss cannot yet compare with that from polarization behaviors because the *ε*_p_′′, especially in the second half frequency range, is ≈70–100 times higher than *ε*_c_′′ (Figure 3a). This is also firmly validated by the highly frequency‐sensitive *ε*′′ together with the Cole–Cole plots that display multiple distorted semicircles without the presence of tail lines (Figure 3 h).^[^ ^48^ ^]^ Therefore, it is reasonably deduced that the ever‐growing *ε*′′ mostly originates from the strong polarization loss induced by the N dopant and sp^2^─sp^3^ C─C bonds.

The coordination with N moieties imparts a heterogeneous electric distribution to the carbon framework, suggesting a higher possibility of arousing polarization. Based on the first‐principles density functional theory (DFT) calculations, the electrostatic potential (ESP) map of the carbon rings‐triazine units conjugated structure shows that the electron‐rich region is located at the unconjugated edge neighboring N atoms of the triazine domain, while the electron‐deficient region was distributed around nearly the whole sp^2^ carbon rings (Figure 3f). Such a distribution will lead to the noncoincidence of the negative and positive charge centers and thus constitute an electric dipole. Moreover, the Mulliken charges of N, sp^3^ C, and sp^2^ C atoms in the dotted squares are −0.356, 0.437, and −0.101, 0.147, respectively. This not only demonstrates that electric dipoles are formed between N and C atoms but also points out that sp^3^ C possesses a relatively larger potential to engender stronger electric dipoles with N than sp^2^ C due to the nature of weak electronegativity of sp^3^ C. The results of the DFT calculation show that a large molecular dipole moment of 8.73 D is formed for such a local polarization domain, and the bond dipole moments between N and sp^3^ C and between N and sp^2^ C are 0.71 D and 0.22 D, respectively. The presence of abundant dipole centers suggests that considerable dipolar polarization loss ought to be raised under the alternating EM condition for AC‐CN550. However, the benign *ε*′′ clearly denies this possibility. This can be explained by the polarization cancellation caused by the over‐high concentration of polarization units. One consensus is that the sp^3^ carbon domains always spatially involve N sites, thereby, given the high content of N moieties as well as the abundance of sp^3^ carbon clusters in AC‐CN550, the dipolar moments of the numerous polarization units distributed throughout the framework, which are more likely to constitute a symmetric plane, will lead to a weak total vectorial sum, that is, the polarization cancellation. This effect can be easily understood by the fact that the graphitic carbon nitride (g‐C_3_N_4_) that is completely composed of C─N sp^2^‐hybridized bonds exhibits an ultrasmall dipole moment of only 0.045 D.^[^ ^49^ ^]^ We also measure the EM parameter of g‐C_3_N_4_, and the results show that the *ε*′′ is even no higher than 0.4 (Figure S13, Supporting Information), manifesting the negligible dipolar polarization loss ability under the EM field. Likewise, the mechanism is also applicable to the interpretation of the poor dielectric loss capacity for AC‐CN550, as well as AC‐CN650.

**Figure 4:**
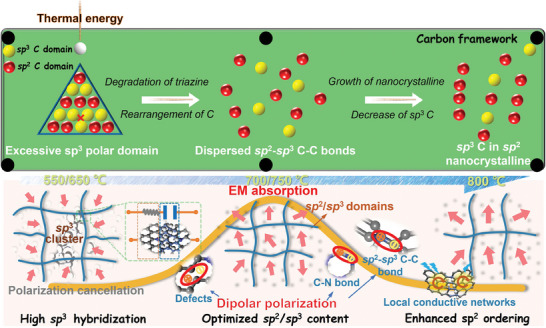
The schematic illustration is based on the “billiard ball” model, and showing the correlation between the structural evolution of AC‐CN nanosheets and their dielectric loss behaviors.

Enhancing pyrolysis temperature results in a continuous process of triazine's degradation together with a sp^2^─sp^3^ hybridization evolution, in which the loss of N atoms is much faster than the transition of sp^3^ C. For AC‐CN750, the N/C ratio is as low as ≈21.86% while the sp^3^/sp^2^ carbon fraction is still up to ≈53.40%. This phenomenon indicates that the EM loss contribution from N species is limited by its content. Meanwhile, the more ordered sp^2^─sp^3^ hybridized structure also means that the electric dipoles induced by sp^3^ C and N atoms become the main role in polarization loss owing to the absence of triazine‐based polarization unit in the AC‐CN nanosheets prepared above 700 °C. As shown in Figure 3g, the ESP map shows a conspicuous potential gap between sp^2^ and sp^3^ carbons with the electrons more biased in favor of sp^2^ carbons, firmly manifesting that electric dipoles can be formed with an orientation from sp^2^ C to sp^3^ C as the result of the stronger electronegativity of sp^2^ C atoms. This is also reflected in the Mulliken charges of the connected sp^2^ and sp^3^ carbons, which are −0.216 and 0.138, respectively. A severe lattice distortion at this region is revealed in the optimized structure (Figure S14, Supporting Information), where the moment of the dipole along the sp^2^─sp^3^ C─C covalent bond is calculated as 0.32 D with direction sp^2−^→^+^sp^3^, and the sp^3^ N─sp^3^ C bond can afford a dipole moment of 1.14 D. Nonetheless, because of the negative contribution from the adjacent sp^2^ C atom on the polarization of central N atom, the resultant N induced electric dipole is actually deteriorated, which shows a sharply reduced moment of 0.51 D. On contrary, this issue does not exist for sp^2^ C atom of the sp^2^─sp^3^ C─C bonds because polarization cannot be aroused between two sp^2^ C atoms, and the another adjacent sp^3^ C atom can even further provide a strengthened dipole vector, increasing the dipole moment to 0.74 D. Combining the higher atomic content with the larger dipole, we can infer that sp^3^ carbons play a more dominant role in raising dipolar polarization than N atoms in the as‐prepared AC‐CN nanosheets. Moreover, the highly disordered sp^2^─sp^3^ domains are very instrumental in preserving the sp^3^ C‐induced polarization intensity by avoiding polarization cancellation. For a better understanding, the effect of the thermal‐driven structural evolution on the polarization behaviors of AC‐CN nanosheets can be illustrated vividly by a “billiard ball” model, as depicted in **Figure** **4**. For the carbon rings‐triazine units conjugated structure, the excessive content of N species‐containing sp^3^ C polar domain constitutes a symmetric distribution within the disordered framework, which leads to a weak total polarization; ii) By right of the decomposition of triazine together with the rearrangement of C, the preserved sp^3^ C domains are randomly dispersed within the carbon plane to engender the electric dipoles between sp^2^ and sp^3^ carbons, thereof leading to an enhanced dipolar polarization; iii) with the continuous transition of carbon atoms, the residual sp^3^ C domains become interspersed within the extensive sp^2^ nanocrystalline, in which sp^3^ C induced dipolar polarization still dominates the dielectric loss behaviors. Therefore, by virtue of the unparalleled contribution from the sp^2^─sp^3^ C─C induced electric dipoles, intense dielectric relaxations can be triggered to convert the EM energy into Joule heat efficiently when radiated by the external EM waves.

### 2.4 EM Absorption Performance of AC‐CN Nanosheets

**Figure 5:**
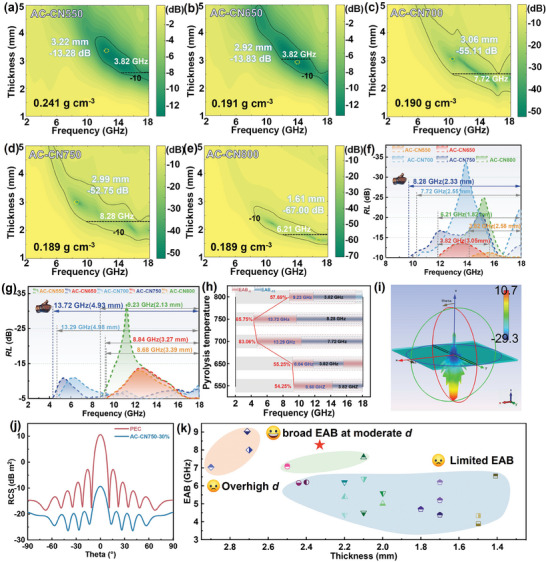
a–e) 2D contours of RL versus frequency at the varying thickness of 1–5 mm of AC‐CN nanosheets. f) EAB_10_, g) EAB_5_, and h) the corresponding frequency range summary. i) 3D radar wave scattering signal and j) RCS simulation curves of the model with an absorption layer filled with AC‐CN750 coated on a PEC base. k) Comparison of EAB_10_ and the matching thickness with those of the recently reported advanced carbon‐based absorbers.

The dependence of the EM absorption performance of AC‐CN nanosheets on their structural evolution was investigated on the basis of transmission line theory.^[^ ^50^, ^51^ ^]^ The results indicate that all the AC‐CN nanosheets exhibited significantly enhanced EM absorption performance in both RL and EAB compared to AC750 (Figures S15 and S16, Supporting Information). This preliminarily manifests that the constructing sp^2^─sp^3^‐hybridized structure is able to greatly boost the attenuation capacity of the carbon framework. At the same time, the AC‐CN nanosheets obtained over 700 °C outperform AC‐CN550 and AC‐CN650 prominently, which is demonstrated not only by their more than 4 times higher RL but also by the dramatically extended EAB toward the X band (**Figure** **5**a–e). In this regard, the aforementioned polarization cancellation effect is responsible for the poor EM absorption performance of AC‐CN550 and AC‐CN650. As a striking contrast, AC‐CN750 presents a broad EAB_10_ (below −10 dB, 90% absorption efficiency) up to 8.28 GHz at a modest thickness of 2.33 mm (Figure 5f), in addition to a strong RL_min_ of −52.75 dB. This EAB_10_ is more than 320% larger than that of the purely sp^2^‐hybridized AC750, which unambiguously demonstrates the huge potential of sp^2^─sp^3^ C─C polar covalent bonds in EM absorption applications. In addition, an intrinsic advantage of lightweight is permitted for this sp^2^─sp^3^‐hybridized carbon absorber, reflected by the small bulk density as low as ≈0.190 g cm^−3^. More impressively, the corresponding EAB_5_ (below ‐5 dB, 70% absorption efficiency) of AC‐CN750 achieves 13.72 GHz at 4.93 mm, nearly covering all of the C, X, and Ku bands (Figure 5g). In other words, the EAB_5_ occupies 85.75% of the most common millimeter‐microwave frequency range (Figure 5h), which is quite astonishing even though the matching thickness is not ideal enough. To our knowledge, this performance upgraded the EAB of carbon EM absorbers at a fixed thickness to a higher level, which may endow the as‐obtained AC‐CN nanosheet with great potential for application in scenarios like civilian anti‐EM wave infrastructure and facilities. Increasing the pyrolysis temperature to 800 °C results in a slight decrease of EAB to 6.21 GHz. In this sample, the dipolar polarization induced by sp^2^─sp^3^ C─C bonds still contributes dominantly to the dielectric loss, while the presence of an increased proportion of sp^2^ C domains leads to an enhanced permittivity and thus the mismatched impedance (Figure S17, Supporting Information). Therefore, tuning the sp^3^/sp^2^ carbon fraction to ≈53.40% is considered an optimized condition to arouse broadband EM absorption. Furthermore, the frequency domain‐based radar cross‐section (RCS) simulation was implemented under a far field to demonstrate the functionality of the as‐synthesized AC‐CN nanosheet (Figure S18, Supporting Information). As seen in Figure 5i,j, the AC‐CN750‐filled absorbing layer efficiently reduces the reflection of EM wave to more than −10 dB m^2^ at the surface of the perfect electric conductor (PEC) base over the whole incident angle. Notably, when the incident angle is adjusted to ≈±10° or larger, the RCS decreases to −20 dB m^2^, comparable to that of a bird. Compared to the recently reported state‐of‐the‐art carbon‐based absorbers, the AC‐CN750 synthesized in this study not only possesses a unique light‐element‐only feature but also demonstrates a significant advantage in possessing a much wider EAB at a moderate thickness (Figure 5k; Table S3, Supporting Information). These promising results highlight the potential of the as‐synthesized AC‐CN nanosheets for practical applications in broadband EM elimination.

## 3 Conclusion

In summary, a 2D carbon nanosheet material that contained sp^3^‐hybridized carbons of high content successfully stabilized in the sp^2^ domains is developed, enabling high‐performance EM absorption over an extended frequency range. This is achieved by customizing and selectively engineering N moieties incorporated within the sp^2^─sp^3^‐hybridized carbon framework, using a facile and mild salt‐assisted thermal‐polymerization and pyrolysis strategy. A distinctive sp^2^─sp^3^ C─C dipolar polarization behavior is aroused by the polar covalent bonds between sp^2^ carbon and sp^3^ carbon atoms. With the microstructural conversion from the carbon rings‐triazine units conjugated plane to the N‐doped carbon skeleton, the sp^3^/sp^2^ carbon fraction decreases from 94.42% to 45.35%. The excessive content of sp^3^ carbons and N species leads to a polarization cancellation effect and thus impairs the dielectric loss; while adjusting the sp^3^/sp^2^ carbon fraction to an optimized level (≈53.40%) endows the as‐prepared AC‐CN nanosheet with remarkable dielectric loss capability. Supported by DFT calculations, the resulting sp^2^─sp^3^ C─C dipolar polarization contributes dominantly to the dielectric loss, which gives rise to an extended EAB_10_ of 8.28 GHz (at 2.33 mm) and an ultrawide EAB_5_ of 13.72 GHz (at 4.93 mm). This study opens up alternative perspectives for the design of carbon EM absorbers by constructing polar covalent C─C bonds between the carbon atoms of different hybridizations to afford electric dipoles. The application of this sp^2^─sp^3^ C─C hybridized nanosheet to wide‐band EM elimination broadens the new horizon in carbon absorbers and provides pioneering enlightenment on the high‐precision regulation revolution of the advanced EM absorption materials.

## Conflict of Interest

The authors declare no conflict of interest.

## Supporting information

Supporting Information

## Data Availability

Research data are not shared.
