# Supplementary material for: Unveiling the sp2─sp3 C─C Polar Bond Induced Electromagnetic Responding Behaviors by a 2D N‐doped Carbon Nanosheet Absorber
Source: Adv Sci (Weinh). 2023 Dec 3;11(4):2306159. doi: 10.1002/advs.202306159 (PMC10939080; doi:10.1002/advs.202306159)
Supplement: Supplementary file 1 — Supporting Information [file ADVS-11-2306159-s001.pdf]

ADVANCED SCIENCE
Open Access

Supporting Information

for *Adv. Sci.*, DOI 10.1002/advs.202306159

Unveiling the sp^2—sp^3 C—C Polar Bond Induced Electromagnetic Responding Behaviors by a 2D N-doped Carbon Nanosheet Absorber

Can Zhang, Jian-Tang Jiang, Zhenjie Guan*, Yuanyuan Zhang, Yining Li, Bo Song, Wenzhu Shao and Liang Zhen*

Supporting Information

Unveiling the sp^2-sp^3 C-C polar bond induced electromagnetic responding behaviors by a 2D N-doped carbon nanosheet absorber

Can Zhang, Jian-Tang Jiang, Zhenjie Guan*, Yuanyuan Zhang, Yining Li, Bo Song, Wenzhu Shao, and Liang Zhen*

Experimental Section

Synthesis of AC-CN nanosheets. In a typical synthesis, 1.0 g resol (provided by ZhengZhou BoRun New Material Co., Ltd.) was absolutely dissolved in 80 ml anhydrous ethanol by ultrasonication for 15 min, followed by adding 3.0 g melamine (purchased from Shanghai Aladdin Biochemical Technology Co., Ltd.) into the solution for another 15 min ultrasonication. The obtained solid-liquid mixture was then heated to 75 °C under continuous magnetic stirring with adding 100 ml deionized water and 15.0 g NaCl (obtained from Shanghai Macklin Biochemical Technology Co., Ltd.). The system was sealed and kept at this temperature for 4 h to ensure the complete and uniform combination of resol and melamine molecules under the hydrogen bonding interaction. After this, the flask was unsealed to ensure the liquid evaporate. Subsequently, the dried solid product was ground uniformly to get a homogeneous precursor. The obtained powder was then annealed in a tube furnace under an Ar atmosphere by following the below procedure: heat to 400 °C at 5 °C min^{-1} and hold for 2 h for the polymerization between melamine and resol; heat to 500 °C at 2 °C min^{-1} and hold for 1 h for the formation of melem units; heat to 550-800 °C at 2 °C min^{-1} and hold for 3 h for the microstructure modulation. The products were denoted as AC-CN*x*, where *x* represents the pyrolysis temperature (550, 650, 700, 750, and 800 °C). Finally, the as-prepared carbon nanosheet was repeatedly washed with deionized water to get rid of NaCl and a subsequent drying at 60 °C. For comparison, resol was

solely treated in the same route at a pyrolysis temperature of 750 °C, and the resultant bare carbon product was named AC750.

Material Characterizations. The X-ray diffraction (XRD) patterns were collected on a PANalytical X'Pert PRO diffractometer with a Cu Kα radiation source. Fourier transform infrared (FTIR) spectrum was carried out with a Thermo Scientific NICOLET iS10 FTIR spectrophotometer. The X-ray photoelectron spectroscopy (XPS) and the X-ray Auger-electron spectroscopy (XAES) were performed in a ThermoFischer ESCALAB 250Xi spectroscopy using an Al-kα monochromatic excitation source. The visible resonant Raman spectra (vis-Raman) and photoluminescence (PL) spectra were recorded on a Renishaw in-Via spectrometer with a 532 nm laser, and the Ultraviolet Raman spectra (uv-Raman) were conducted on an Andor SR-500i Raman spectrometer with a 325 nm laser. The transmission electron microscope (TEM) study, including the related characterization such as scanning TEM-electron energy loss spectroscopy (STEM-EELS) was conducted using an aberration-corrected Titan G2 microscope operated at 300 kV. The content of sp^2 and sp^3 carbons was calculated by the integrated area based on the two-window method, as follows:

$$\frac{sp^2}{sp^2+sp^3} = \frac{\frac{I_{\pi^*}^{a}}{I_{a^*}^{a}+I_{\sigma^*}^{a}}}{\frac{I_{\pi^*}^{g}}{I_{\pi^*}^{g}+I_{\sigma^*}^{g}}} \qquad \text{(Equation S1)}$$

where the I_s represent the integrated intensities of the 1s→π* transition peak and the 1s→σ* transition peak, respectively. The reference sample of glass carbon is denoted as g, while a refers to the as-prepared AC-CN nanosheets. The atomic force microscopy (AFM) images were obtained by a Bruker Dimension Icon microscopy. Electrochemical impedance spectroscopy (EIS) was recorded using a conventional three-electrode system in 3.5 wt% NaCl solution on a CHI 660E electrochemical workstation (scanning speed 0.001 V/s, frequency range of 10^5 Hz–0.1 Hz). In a typical method, the working electrode is fabricated with the carbon cloth (4.6 mm*4.4 mm) serving as supporter, and the auxiliary electrode and reference electrode are

platinum foil and saturated calomel, respectively. The electron paramagnetic resonance (EPR) was performed on a Bruker EMX plus under the temperature of 77K, with the frequency of 9.84 GHz and the center field of 3410 G.

Electromagnetic Measurements. The electromagnetic parameters were measured using an Agilent N5230A vector network analyzer (VNA) in the 2-18 GHz frequency range. To prepare the test specimen, the samples were uniformly mixed with paraffin wax at a mass ratio of 30% and then compacted into a coaxial ring with an outer diameter of 7.00 mm and an inner diameter of 3.04 mm. The reflection loss (RL) was calculated based on the transmission line theory as follows:

$$\mathrm{RL}=20\log\left|\frac{(Z_{\mathrm{in}}-Z_0)}{(Z_{\mathrm{in}}+Z_0)}\right| \quad \text{(Equation S2)}$$

$$Z_{\mathrm{in}}=Z_0\sqrt{\frac{\mu_{\mathrm{r}}}{\varepsilon_{\mathrm{r}}}}\tanh\left[\frac{\mathrm{j}2\pi fd}{c}\sqrt{\mu_{\mathrm{r}}\varepsilon_{\mathrm{r}}}\right] \quad \text{(Equation S3)}$$

in which Z_{in} and Z_0 successively represent the input impedance of the absorber and the impedance of free space, d is the thickness of absorber, and c is the velocity of light in vacuum.

theoretical calculations

Radar cross-section (RCS) simulation based on Frequency domain. The RCS performance of AC-CN nanosheets was studied by a frequency domain-based simulation on CST Studio Suite 2019 to investigate their promising practical applications. The numerical simulation was accomplished by calculating the following formula:

$$RCS(dB\ m^2) = 10log\left(\left(\frac{4\pi S}{\lambda^2}\right)|E_S/E_i|^2\right) \quad \text{(Equation S4)}$$

where S and λ represent the area of the absorber and the wavelength of electromagnetic wave, respectively, and E_s and E_i successively are the electric field intensity of transmitting waves and the electric field intensity of the receiving wave. In the simulation model, the electromagnetic absorption layer (180mm*180mm*2.5mm) sticks to a perfect electric conductor (PEC, 180mm*180mm*0.5mm) layer with open boundary conditions, and the incident

electromagnetic wave (9.00 GHz) penetrates the upper absorption layer along the *z-axis*. The setting of the length and width is accordance with the standard size of the template plates for the arch method to measure electromagnetic absorption performance, the thickness of absorption layer is dependent on the matching thickness of the as-prepared AC-CN nanosheets.

DFT computational Details. The geometry optimizations were performed with ωB97X-D/def2SVP level of theory. Harmonic vibration frequency calculations were performed for all stationary points to confirm them as a local minimum at the same theoretical level. The single-point energy (SP) calculations were performed on the optimized geometries at ωB97X-D3(0)/def2-TZVPPD theoretical level. The geometric optimization and subsequent frequency analysis were carried out by Gaussian 16 programs and the SP calculations were carried out by the ORCA 5.0.3. The analyses of dipole moment and electrostatic potential (ESP) on molecular vdW surface were finished by Multiwf. The ESP map was rendered by VMD program based on the outputs of Multiwfn. The component of the dipole moment of the whole molecule can be computed as

$$\mathbf{D}_F = \sum_{A\in F}[Z_A\mathbf{R}_A - \int w_A(\mathbf{r})\rho(\mathbf{r})\mathbf{r}\,\mathrm{d}\mathbf{r}] \qquad \text{(Equation S5)}$$

where **R** is the position of the nucleus, Z is the nuclear charge, $\rho(\mathbf{r})$ is the electron density, and $w(\mathbf{r})$ is the atomic space partition function.

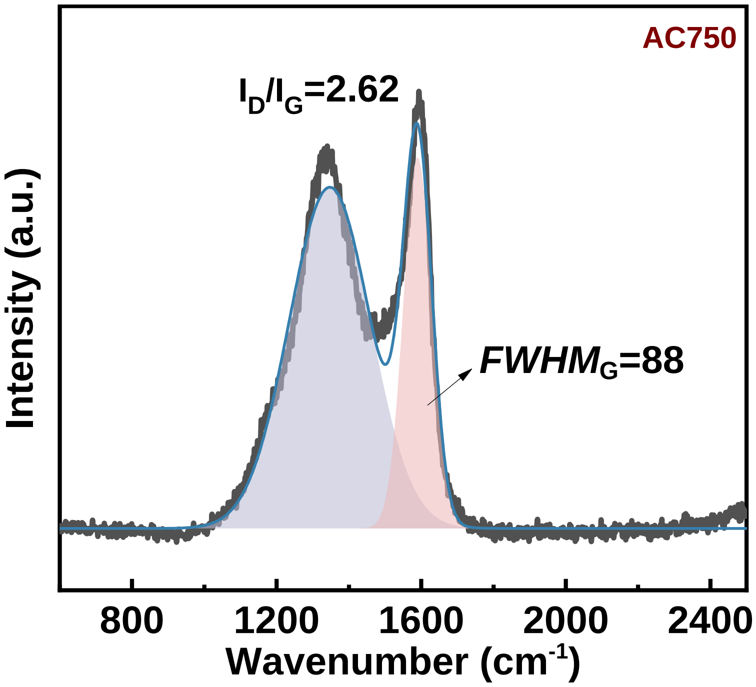

Figure S1. Vis-Raman spectra of the control sample (AC750), which shows a much weaker dispersion of the G band.

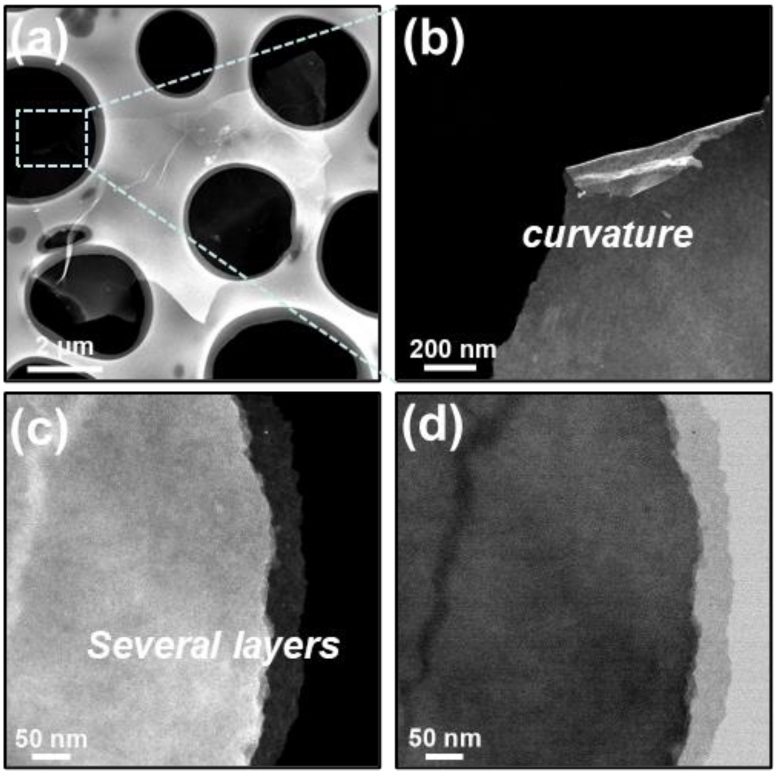

Figure S2. a, b, c) HAADF TEM images and d) bright-field TEM image of AC-CN750, where a multilayer stack of nanosheets is clearly seen.

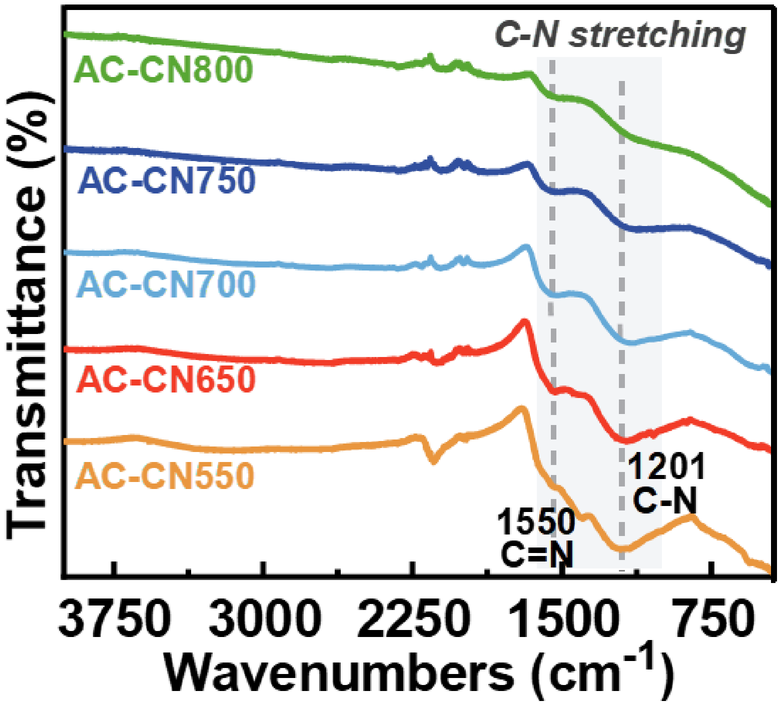

Figure S3. FTIR spectra of AC-CN nanosheets, where the C-N stretching region originates from the triazine rings,[1] and the diminished C-N stretching with the elevated pyrolysis temperature indicates the gradual degradation of triazine units.

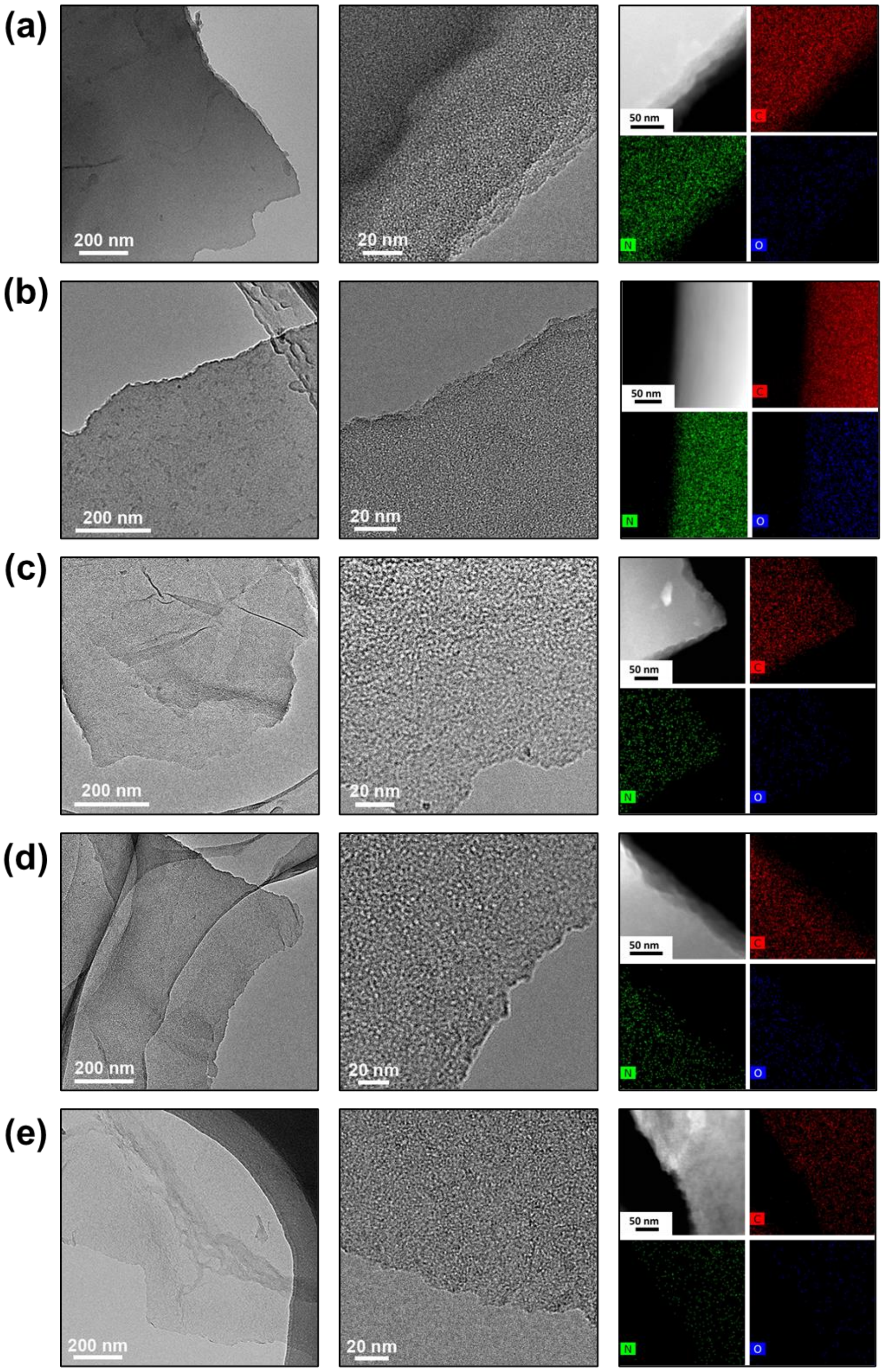

Figure S4. TEM images and EDS images of a) AC-CN550, b) AC-CN650, c) AC-CN700, d) AC-CN750, and e) AC-CN800, which demonstrate that the 2D nanosheets survive the elevated pyrolysis temperature, and C and N elements distribute within the nanosheets uniformly.

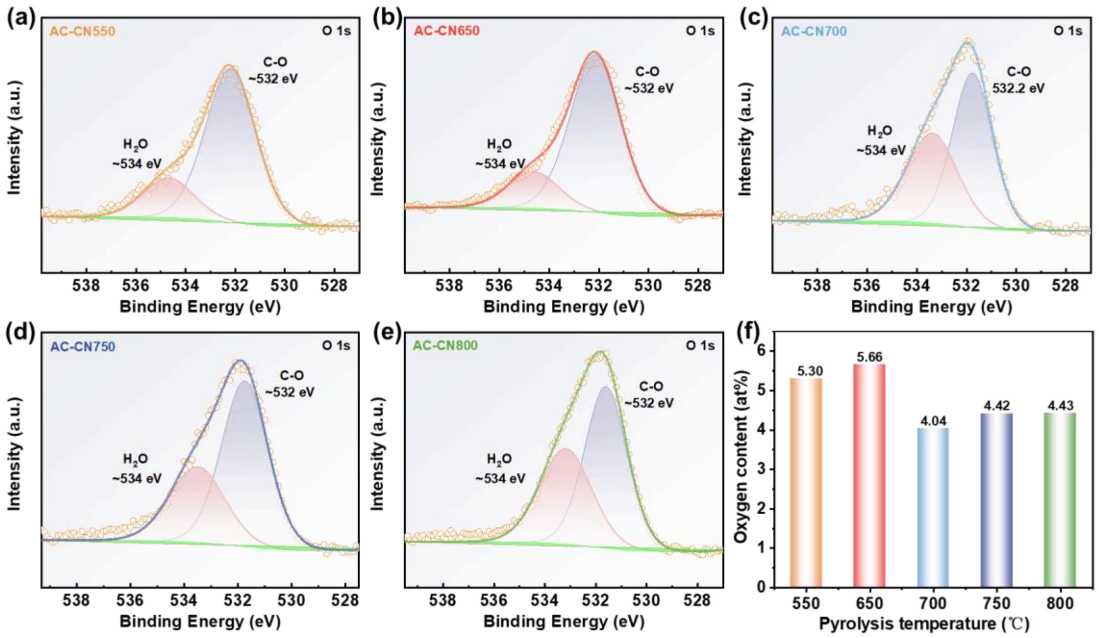

Figure S5. High-resolution XPS spectra of O 1s of a) AC-CN550, b) AC-CN650, c) AC-CN700, d) AC-CN750, and e) AC-CN800, and f) their oxygen content excluding O from H_2O, showing that the configuration of these low-content O atoms remains unchanged.

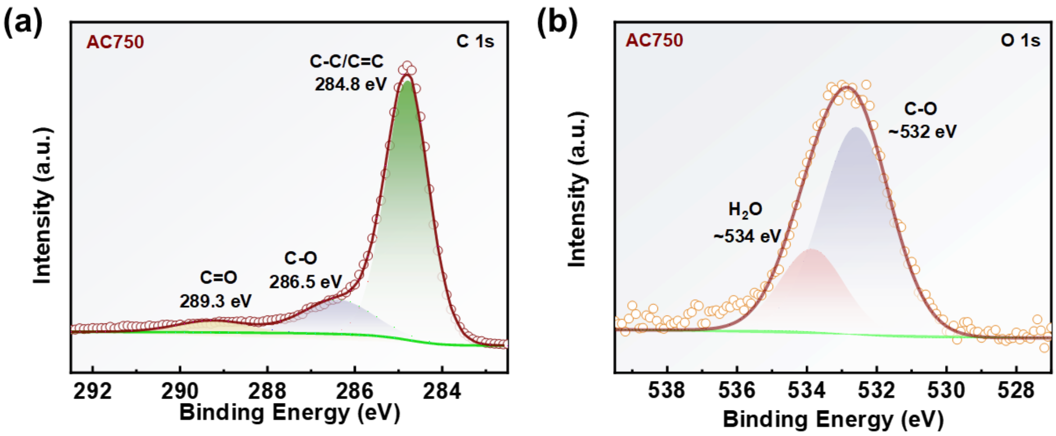

Figure S6. High-resolution XPS spectra of a) C 1s and O 1s of AC750, which demonstrates the distinct chemical environment compared to AC-CN nanosheets.

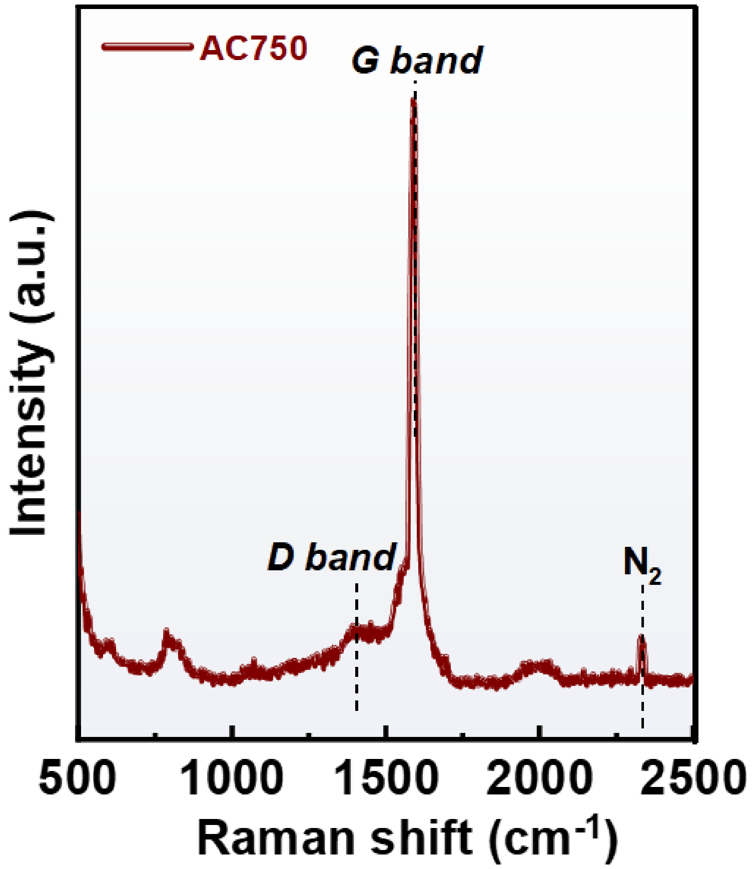

Figure S7. Uv-Raman spectra of AC750, where no T band is exhibited but an obvious D band together with an intense G band are still present.

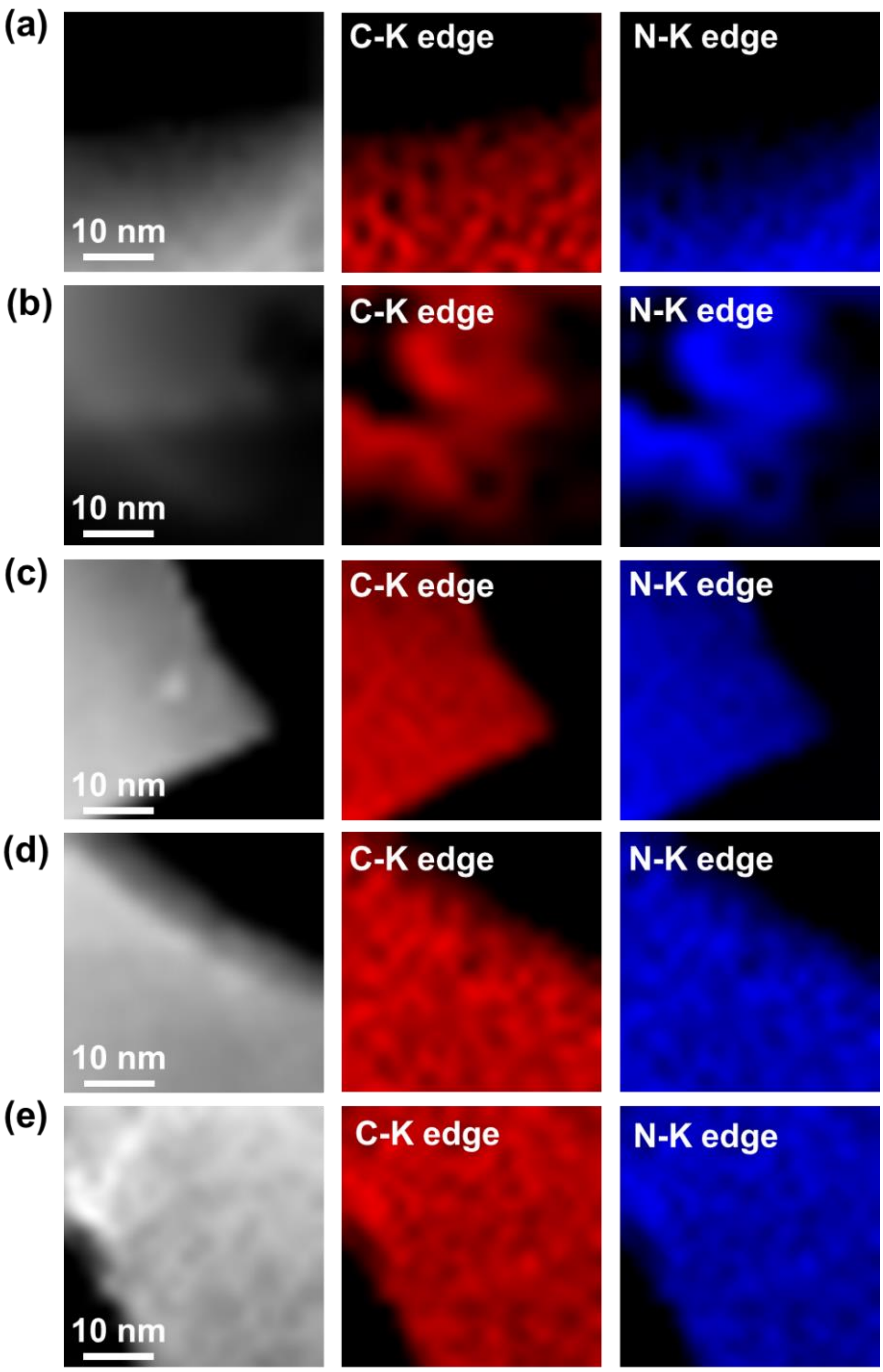

Figure S8. Core-loss spectrum images and the EELS mappings of C-K edge and N-K edge of a) AC-CN550, b) AC-CN650, c) AC-CN700, d) AC-CN750, and e) AC-CN800, from which the uniform distribution of C and N atoms is firmly demonstrated.

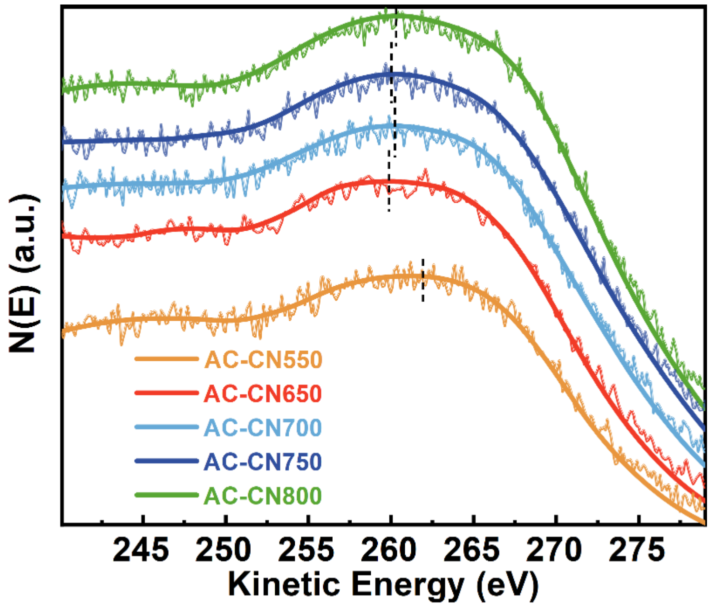

Figure S9. XAES spectra of AC-CN nanosheets, from which the dN/dE spectra are derived.

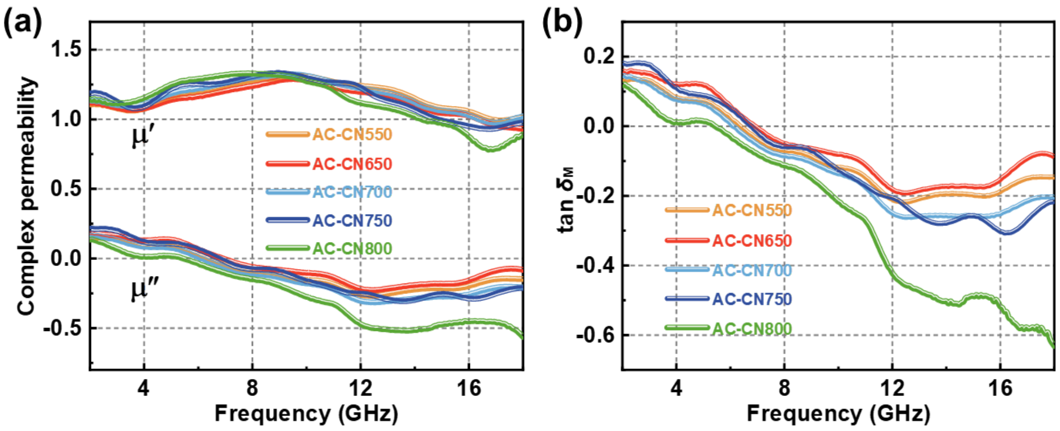

Figure S10. a) Frequency dependence of permeability and the b) magnetic loss factor (tan δ_M) of AC-CN nanosheets, in which the negative tan δ_M factor over the majority of frequency indicates a negligible magnetic loss.

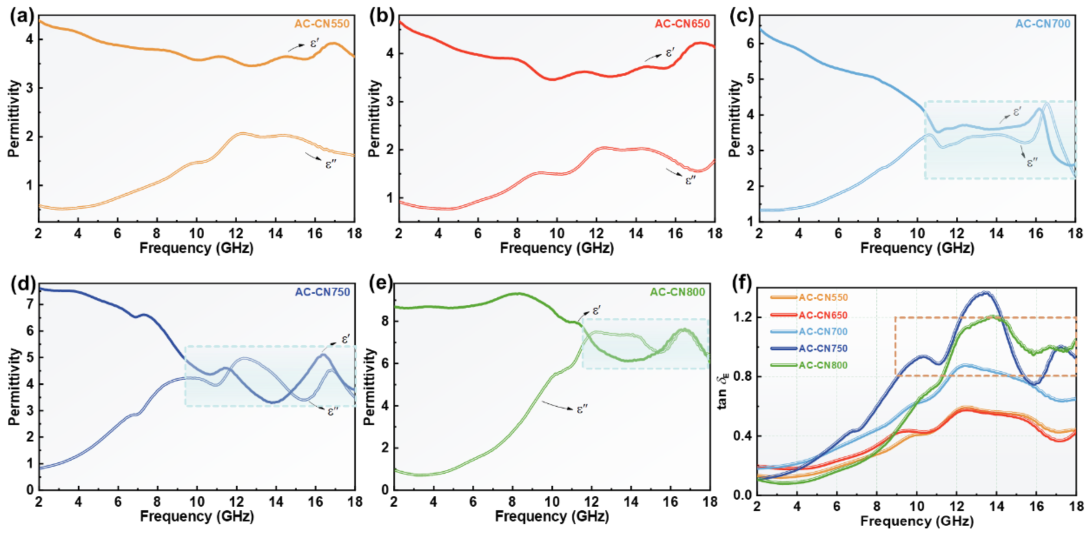

Figure S11. Frequency dependence of a-e) complex permittivity and f) dielectric loss factor (tan δ_E) of AC-CN nanosheets, where AC-CN750 shows the best matching between ε' and ε'' within the widest frequency range.

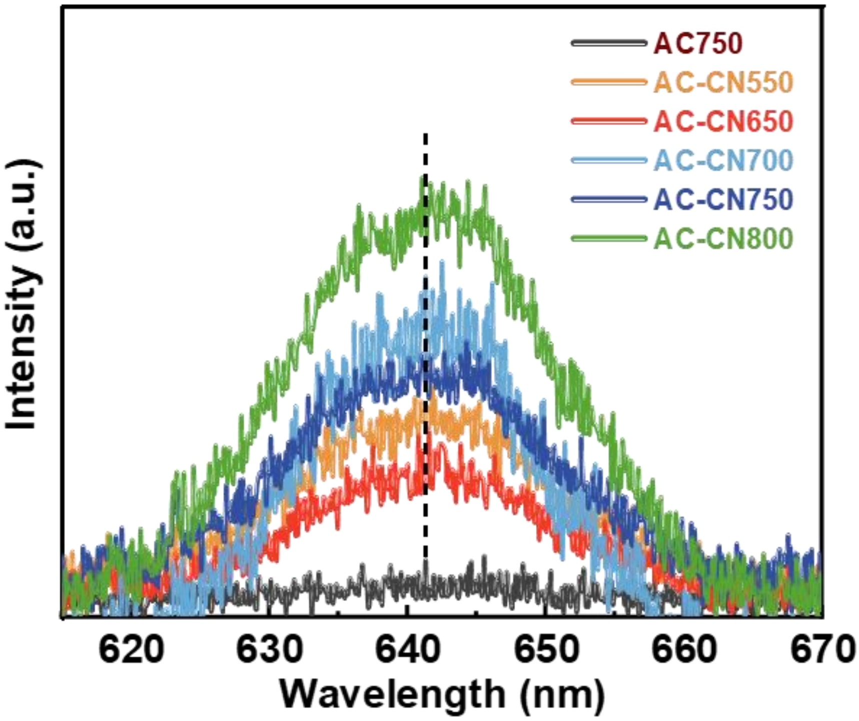

Figure S12. PL spectra of AC-CN nanosheets, where AC750 shows no PL peak but AC-CN nanosheets display a generally increasing PL intensity at ~642 nm, revealing that the PL signal comes from the induced defects within the carbon framework and the content of defects decreases with the enhancing pyrolysis temperature. The possibility of this peak originating from the Raman signal is firstly excluded.

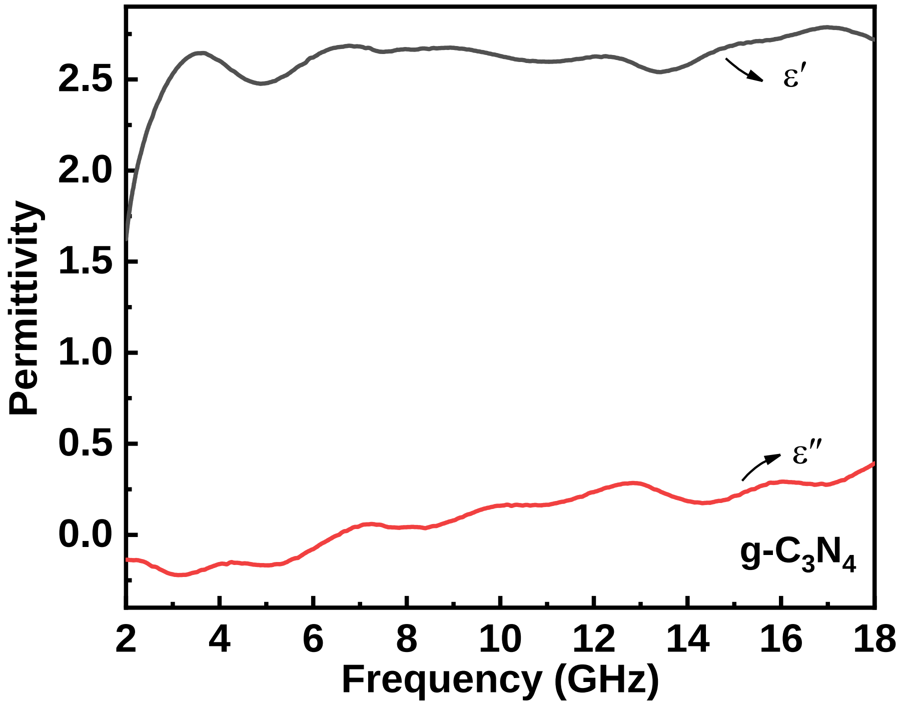

Figure S13. Frequency dependence of permittivity of g-C3N4, which indicates negligible dielectric loss ability.

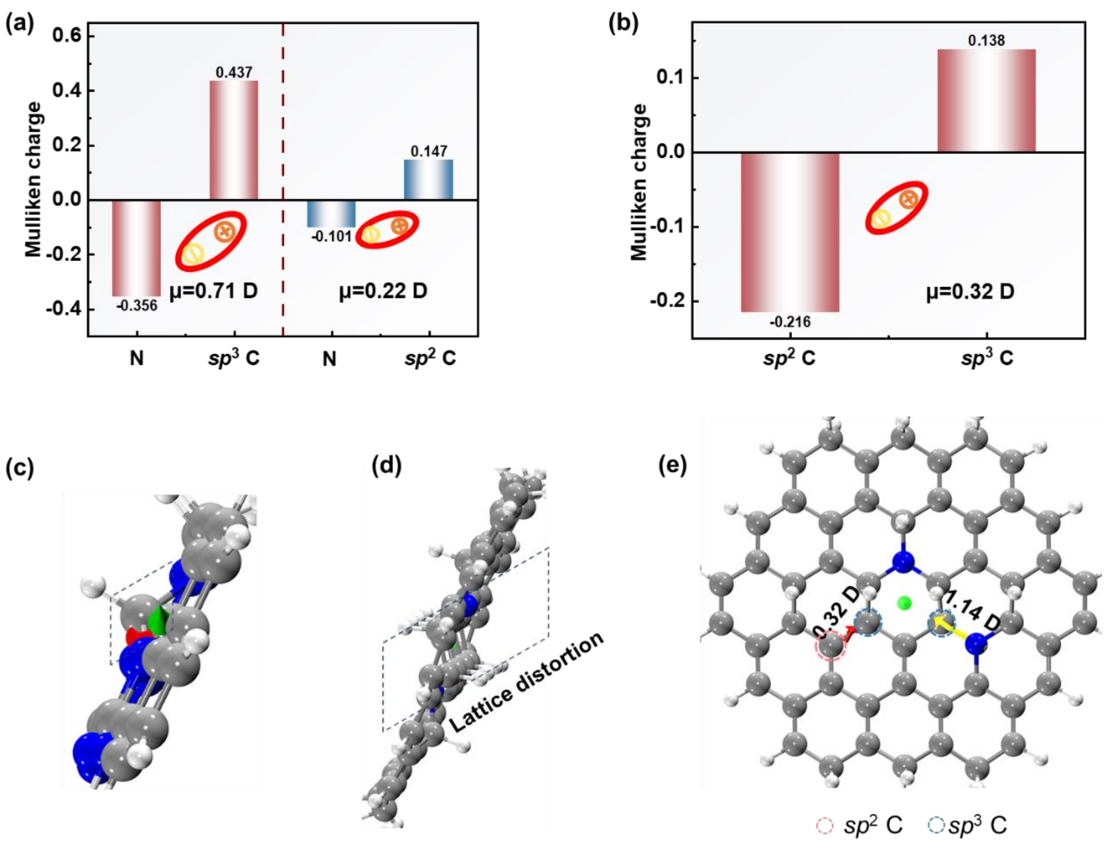

Figure S14. a) Mulliken charge of N, sp3 C and sp2 C atoms of the bond between them in the carbon ring-melem conjugated structure, and c) the side view of the corresponding model; b) Mulliken charge of sp3 C and sp2 C atoms of the sp3-sp2 C-C bond in the more ordered hybrid structure with sp3 carbons incorporated within sp2 domain, and d) the side view of the corresponding model in DFT calculations, where severe lattice distortion generated by sp3 carbon is seen. e) the dipole formed along the polar bonds between sp2 carbon and sp3 carbon atoms, and between the N atom and sp3 carbon atom.

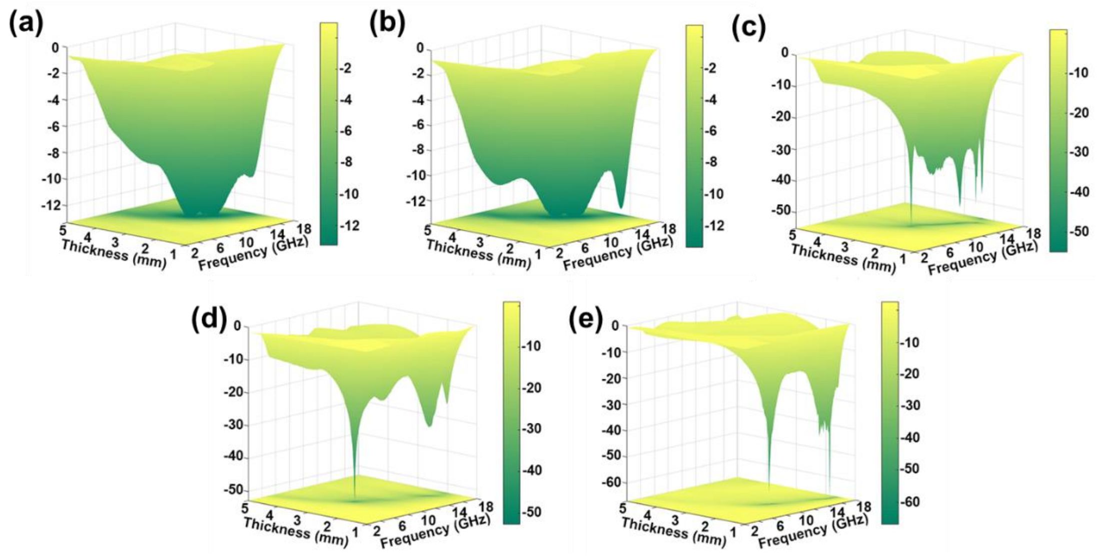

Figure S15. 3D map of reflection loss-frequency-thickness of a) AC-CN550, b) AC-CN650, c) AC-CN700, d) AC-CN750, and e) AC-CN800.

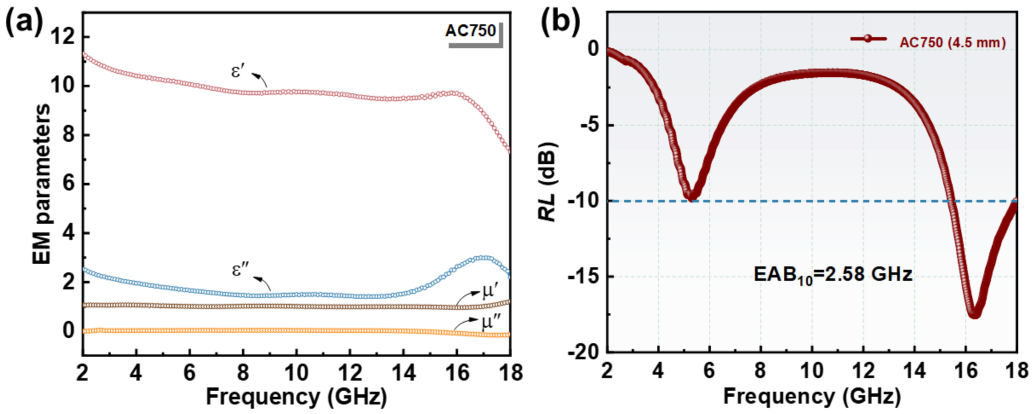

Figure S16. a) Frequency dependence of electromagnetic parameters and the b) effective absorption bandwidth of the control sample AC750, demonstrating its unsatisfactory performance.

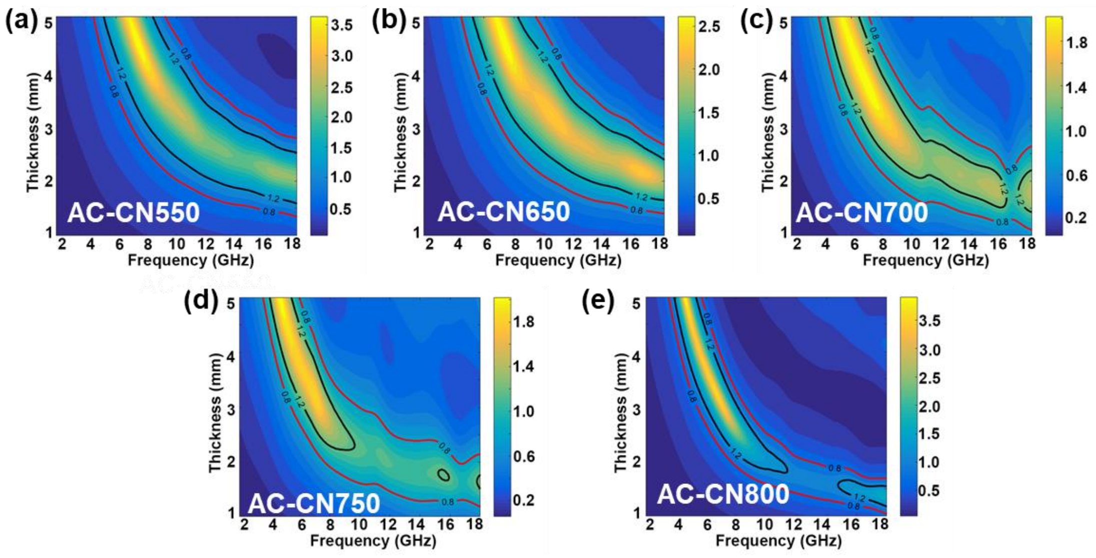

Figure S17. a-b) The 2D contour of the impedance matching characteristic $Z=|Z_{in}/Z_0|$ for AC-CN nanosheets, where AC-CN750 exhibits largest impedance matching region with a Z located within 0.8-1.2.

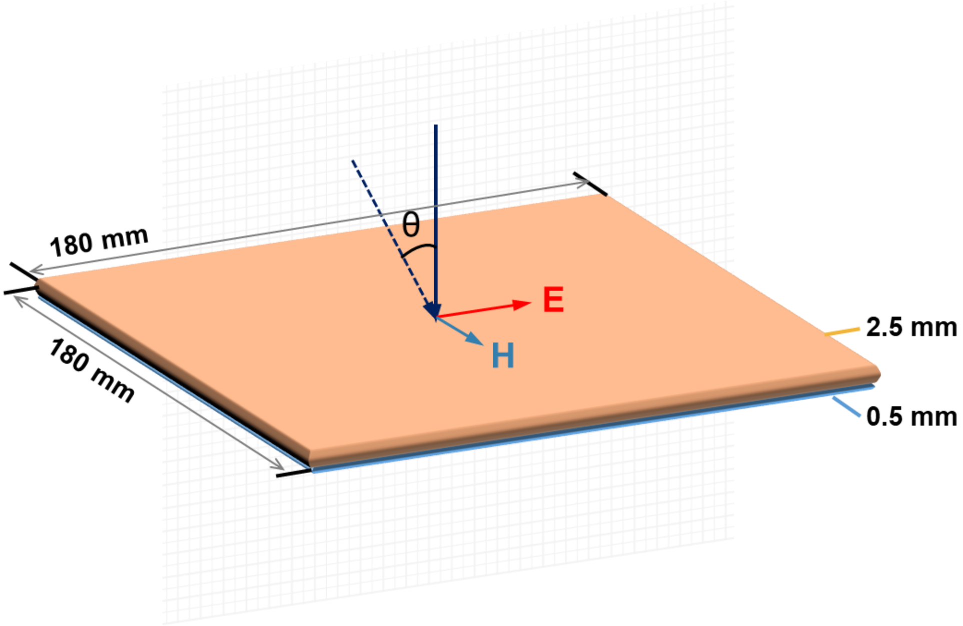

Figure S18. The model employed in the RCS simulation, in which the absorption layer with a thickness of 2.5 mm was coated on a PEC base of 0.5 mm.

Table S1 Parameter D of XAES spectra, I_D/I_G of vis-Raman spectra, and sp^3/sp^2 carbon fraction derived from EELS

Sample	Parameter D (XAES)	I_D/I_G (vis-Raman spectra)	sp^3/sp^2 carbon fraction (EELS)
AC-CN550	15.20	2.53	94.42%
AC-CN650	15.40	2.51	67.22%
AC-CN700	16.40	2.73	60.57%
AC-CN750	17.70	3.18	53.40%
AC-CN850	17.80	3.64	45.35%

Table S2 N/C fractions derived from XPS and EELS spectra

**SAMPLE**	**XPS**			**EELS**		
	IA[a)] $_N$	IA_C	N/C (%)	IA_N	IA_C	N/C (%)
**AC-CN550**	28.48	64.86	43.88	29.80	70.20	42.86
**AC-CN650**	23.37	69.74	33.51	26.80	73.20	36.61
**AC-CN700**	19.35	74.27	26.05	18.00	82.00	21.95
**AC-CN750**	16.78	76.76	21.86	16.20	83.80	19.33
**AC-CN850**	13.64	79.32	17.20	15.90	84.10	18.91

[a)](Integrated Area)

Table S3 EM performance comparison between the AC-CN nanosheets prepared in this study with the recently reported state-of-the-art carbon-based absorbers

SYSTEM	RL (dB)	EAB$_{10}$ (GHz)	*d* (mm)	Ref.
N, S doped GF/GC	-45.64	4.35	1.50	[2]
Co_3O_4@WSe_2-MWCNTs	-56.9	6.56	1.41	[3]
Ni-SA/HPCF	-53.2	5.00	2.00	[4]
NHCS@NiO/Ni	-44.04	4.38	1.70	[5]
Fe@NCNs	-64.75	6.20	1.70	[6]
CF@MXene@MoS_2	-61.51	7.60	2.10	[7]
CoNi@G@NCNTs	-54.00	5.20	1.70	[8]
HPCMCs	-60.70	3.90	1.50	[9]
Co-rGO aerogel	-40.57	7.12	2.50	[10]
Graphene/Si_3N_4	-22.80	8.00	2.70	[11]
FCN-Mo_2C	-36.80	7.04	2.90	[12]
PMPC	-58.60	6.20	2.40	[13]
ZnO/C	-41.70	5.60	2.00	[14]
PC	-32.03	6.60	2.20	[15]
RGO/CNFC aerogel	-70.44	6.16	2.44	[16]
Air@G-Fe/C	-62.70	6.40	2.10	[17]
N-doped RGO aerogel	−39.39	4.70	1.80	[18]
NC@Co/NC	-52.50	4.40	2.20	[19]
C@MoO_2/G	-35.40	4.50	2.10	[20]
C/MnO@void@C	-53.20	5.40	2.20	[21]
N, S, O doped PC	-64.05	9.03	2.71	[22]

Ni-SAs/NC	-36.40	7.08	2.50	[23]
AC-CN700	-55.11	7.72	2.55	
AC-CN750	-52.75	8.28	2.33	Here
AC-CN800	-67.00	6.21	1.82	

References

[1] W. Niu, K. Marcus, L. Zhou, Z. Li, L. Shi, K. Liang, Y. Yang, *ACS Catal.* **2018**, *8*, 1926.
[2] W. Zhong, B. Li, Z. Ma, C. Zhu, F. Yan, X. Zhang, Y. Chen, *Carbon* **2023**, *202*, 235.
[3] Q. Wang, B. Niu, Y. Han, Q. Zheng, L. Li, M. Cao, *Chem. Eng. J.* **2023**, *452*, 139042.
[4] X. Zhang, B. Li, J. Xu, X. Zhang, Y. Shi, C. Zhu, X. Zhang, Y. Chen, *Adv Funct Mater.* **2023**, *33*, 2210456.
[5] B. Li, Z. Ma, X. Zhang, J. Xu, Y. Chen, X. Zhang, C. Zhu, *Small* **2023**, 2207197.
[6] T. Gao, R. Zhao, Y. Li, Z. Zhu, C. Hu, L. Ji, J. Zhang, X. Zhang, *Adv Funct Mater.* **2022**, *32*, 2204370.
[7] J. Wang, L. Liu, S. Jiao, K. Ma, J. Lv, J. Yang, *Adv. Funct. Mater.* **2020**, *30*, 2002595.
[8] D. Guo, H. Yuan, X. Wang, C. Zhu, Y. Chen, *ACS Appl. Mater. Interfaces* **2020**, *12*, 9628.
[9] H. Zhao, X. Xu, Y. Wang, D. Fan, D. Liu, K. Lin, P. Xu, X. Han, Y. Du, *Small* **2020**, *16*, 2003407.
[10] K. Cao, X. Yang, Y. Zhang, J. Wen, J. Chen, X. Hou, R. Zhao, W. Xue, *Carbon* **2023**, *208*, 111.
[11] J. Liang, F. Ye, Y. Cao, R. Mo, L. Cheng, Q. Song, *Adv Funct. Mater.* **2022**, 2200141.
[12] L. Wang, J. Lu, J. Zhang, J. Zhu, *J. Colloid Interface Sci.* **2023**, *641*, 729.
[13] C. Ding, S. Wu, Y. Zhang, Y. Wu, X. Geng, X. Huang, G. Wen, A. Wang, *J. Mater. Chem. A* **2021**, *9*, 16395.
[14] Q. Chen, X. Liu, T. Wang, X. Su, M. Liu, S. Chaemchuen, F. Verpoort, *J. Mater. Sci. Techol.* **2023**, *149*, 255.
[15] Y. Fang, Y. Zhang, W. Wang, S. Wang, X. Hou, Y. Huang, W. Ye, R. Yang, R. Zhao, W. Xue, C. Zhou, H. Zhang, X. He, *Nanoscale* **2023**, *15*, 5855.
[16] R. Zhang, B. Li, Y. Yang, N. Wu, Z. Sui, Q. Ban, L. Wu, W. Liu, J. Liu, Z. Zeng, *Nano Res.* **2023**, *16*, 7931.
[17] C. Wu, J. Wang, X. Zhang, L. Kang, X. Cao, Y. Zhang, Y. Niu, Y. Yu, H. Fu, Z. Shen, K. Wu, Z. Yong, J. Zou, B. Wang, Z. Chen, Z. Yang, Q. Li, *Nano-Micro Lett.* **2023**, *15*, 7.
[18] J. Xu, X. Zhang, H. Yuan, S. Zhang, C. Zhu, X. Zhang, Y. Chen, *Carbon* **2020**, *159*, 357.
[19] P. Liu, S. Gao, Y. Wang, Y. Huang, W. He, W. Huang, J. Luo, *Chem. Eng. J.* **2020**, *381*, 122653.
[20] C. Wu, Z. Chen, M. Wang, X. Cao, Y. Zhang, P. Song, T. Zhang, X. Ye, Y. Yang, W. Gu, J. Zhou, Y. Huang, *Small* **2020**, *16*, 2001686.
[21] P. Xu, R. Zhang, X. Qian, X. Li, Q. Zeng, W. You, C. Zhang, J. Zhang, R. Che, *ACS Appl. Mater. Interfaces* **2021**, *13*, 32037.
[22] J. Tao, L. Xu, C. Pei, Y. Gu, Y. He, X. Zhang, X. Tao, J. Zhou, Z. Yao, S. Tao, H. Wu, *Adv Funct. Mater.* **2023**, *33*, 2211996.
[23] H. Liang, G. Chen, D. Liu, Z. Li, S. Hui, J. Yun, L. Zhang, H. Wu, *Adv Funct. Mater.* **2023**, *33*, 2212604.
